# Optimizing animal models of autoimmune encephalitis using active immunization

**DOI:** 10.3389/fimmu.2023.1177672

**Published:** 2023-07-12

**Authors:** Jenny Linnoila, Negin Jalali Motlagh, Grace Jachimiec, Chih-Chung Jerry Lin, Enrico Küllenberg, Gregory Wojtkiewicz, Rudolph Tanzi, John W. Chen

**Affiliations:** ^1^ Division of Neuroimmunology and Neuroinfectious Disease, Department of Neurology, Massachusetts General Hospital (MGH), Boston, United States; ^2^ Genetics and Aging Research Unit, McCance Center for Brain Health, Department of Neurology, Massachusetts General Hospital (MGH), Boston, MA, United States; ^3^ Department of Radiology, Institute for Innovation in Imaging, Massachusetts General Hospital (MGH), Boston, MA, United States; ^4^ Center for Systems Biology, Massachusetts General Hospital (MGH), Boston, MA, United States

**Keywords:** autoimmune encephalitis, NMDA receptor encephalitis, mouse model, active immunization, experimental autoimmune encephalomyelitis (EAE)

## Abstract

**Background and objectives:**

Encephalitis is a devastating neurologic disorder with high morbidity and mortality. Autoimmune causes are roughly as common as infectious ones. N-methyl-D-aspartic acid receptor (NMDAR) encephalitis (NMDARE), characterized by serum and/or spinal fluid NMDAR antibodies, is the most common form of autoimmune encephalitis (AE). A translational rodent NMDARE model would allow for pathophysiologic studies of AE, leading to advances in the diagnosis and treatment of this debilitating neuropsychiatric disorder. The main objective of this work was to identify optimal active immunization conditions for NMDARE in mice.

**Methods:**

Female C57BL/6J mice aged 8 weeks old were injected subcutaneously with an emulsion of complete Freund’s adjuvant, killed and dessicated *Mycobacterium tuberculosis*, and a 30 amino acid peptide flanking the NMDAR GluN1 subunit N368/G369 residue targeted by NMDARE patients’ antibodies. Three different induction methods were examined using subcutaneous injection of the peptide emulsion mixture into mice in 1) the ventral surface, 2) the dorsal surface, or 3) the dorsal surface with reimmunization at 4 and 8 weeks (boosted). Mice were bled biweekly and sacrificed at 2, 4, 6, 8, and 14 weeks. Serum and CSF NMDAR antibody titer, mouse behavior, hippocampal cell surface and postsynaptic NMDAR cluster density, and brain immune cell entry and cytokine content were examined.

**Results:**

All immunized mice produced serum and CSF NMDAR antibodies, which peaked at 6 weeks in the serum and at 6 (ventral and dorsal boosted) or 8 weeks (dorsal unboosted) post-immunization in the CSF, and demonstrated decreased hippocampal NMDAR cluster density by 6 weeks post-immunization. In contrast to dorsally-immunized mice, ventrally-induced mice displayed a translationally-relevant phenotype including memory deficits and depressive behavior, changes in cerebral cytokines, and entry of T-cells into the brain at the 4-week timepoint. A similar phenotype of memory dysfunction and anxiety was seen in dorsally-immunized mice only when they were serially boosted, which also resulted in higher antibody titers.

**Discussion:**

Our study revealed induction method-dependent differences in active immunization mouse models of NMDARE disease. A novel ventrally-induced NMDARE model demonstrated characteristics of AE earlier compared to dorsally-induced animals and is likely suitable for most short-term studies. However, boosting and improving the durability of the immune response might be preferred in prolonged longitudinal studies.

## Introduction

Anti-N-methyl-D-aspartate receptor (NMDAR) encephalitis (NMDARE) (1), the most common form of autoimmune encephalitis (AE), is a devastating neuropsychiatric disorder characterized by antibodies (Abs) to NMDARs being present in the serum and/or cerebrospinal fluid (CSF) of patients. These Abs bind to NMDARs in the brains of patients and trigger receptor internalization (2), clinically manifesting as psychosis, confusion, seizures, movement disorders, hypoventilation, dysautonomia, and coma. Due to recent advances in the recognition and diagnosis of this disorder, the incidence of AE is increasing and now approximately equals that of infectious encephalitides (3). However, the diagnosis and treatment of AE are currently hampered by an incomplete understanding of its pathogenesis. This is partly due to limited autopsy data (4) and relatively few immune-based studies in patients, such as cytokine profiling (5). As AE remains a comparatively rare disorder, translational animal models are needed to better elucidate its pathophysiology, which can lead to novel diagnostics and therapeutics.

In the first mouse model of NMDARE (6), NMDAR-targeted immunoglobulin G (IgG) from the CSF of NMDARE patients was infused via osmotic pumps into the ventricles of mice. This model demonstrated the direct pathogenicity of patient-derived NMDAR Abs in living mice. However, it did not allow for studies into endogenous processes upstream of Ab appearance. We previously published a post-infectious rodent model of NMDARE (7) where mice made antibodies to NMDARs, but subsequent work revealed that infected mice produce a wide array of autoantibodies, lacking exclusive enrichment for NMDAR Abs (unpublished observation).

NMDAR Abs can be specifically produced by immunizing rodents against the NMDAR. Earlier work revealed that the production of Abs against the GluN1 (NR1) subunit of NMDARs after the oral introduction of an adeno-associated virus (AAV) containing the subunit (8) protected rat brains from glutamatergic excitotoxicity due to induced seizures and strokes. The predominant Abs produced in this model were against a region of GluN1 distinct from that targeted by patients with NMDARE (9, 10). In a different mouse model, Abs produced against proteoliposomes containing tetrameric holoreceptors of GluN1 and GluN2B subunits resulted in more fulminant encephalitis than NMDARE (11), again highlighting the importance of narrower antigen targeting in inducing NMDARE in mice.

Two groups recently published work outlining the active immunization of mice against peptides containing the portion of the amino terminal domain (ATD) of the GluN1 targeted by patients with NMDARE (amino acid residues N368/G369) (12, 13). Curiously, the peptide used by Wagnon et al. (12), GluN1_359-378_, is only 1 amino acid longer than the GluN1_359-377_ peptide used in another study by Ding et al. (13), which failed to produce NMDAR Abs. However, both groups reported that the mice demonstrated changes in their memory and behavior, showing promise as rodent models of NMDARE. While both models were based on experimental autoimmune encephalitis (EAE) (14) methodology, they were widely divergent in terms of technique and timing, raising questions as to which approach to use for subsequent studies. Wagnon et al. examined mice 2 weeks post immunization (12), whereas the other group utilized repeated boosting and examined mice 12 weeks after immunization (13). In these reported models, NMDAR peptide was injected dorsally into mice, into either their shoulder and limb regions (12) or their tail bases (13). In contrast, our group has shown that inducing EAE via subcutaneous ventral surface injections into mouse axillae and inguinal regions (15–18), analogous to techniques described in another study (19), consistently results in illness within a few weeks.

**Figure 1:**
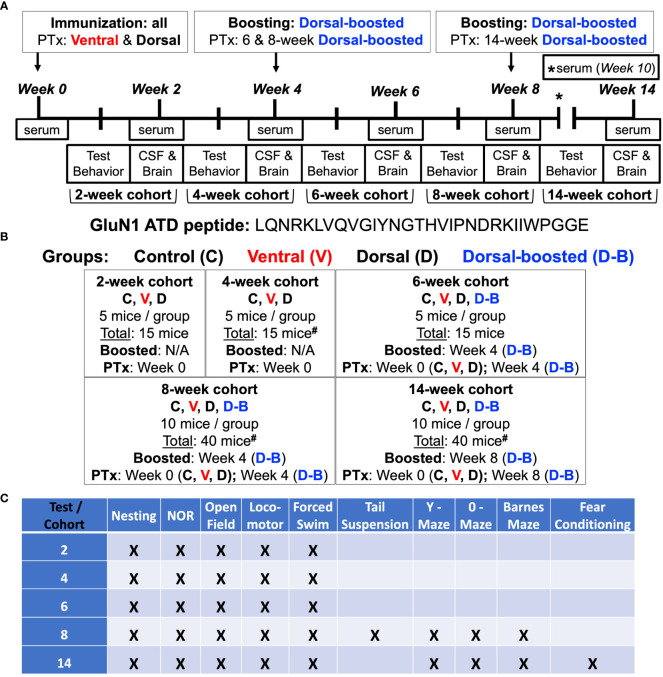
Experimental scheme: **(A, B)** 8-week-old female C57BL/6J mice were injected subcutaneously with an emulsion of complete Freund’s adjuvant (CFA) supplemented with killed *Mycobacterium tuberculosis (Mtb)* and a 30 amino acid peptide (13) flanking the NMDAR GluN1 subunit amino terminal domain (ATD) N368/G369 residue targeted by Abs in patients with NMDARE. Mice were injected with emulsion into either the axillae and groin (Ventral) (15, 18) or on each side of the tail base (Dorsal, Doral-boosted). One group of dorsally-injected mice was reimmunized at 4 and 8 weeks (Dorsal – boosted) (13). Mice were injected with pertussis toxin (PTx) intravenously (Ventral) or intraperitoneally (Dorsal, Dorsal-boosted) at the time of last immunization. Naïve mice were included as controls. There were 5 mice per group in the 2, 4, and 6-week time points and 10 mice per group in the 8 and 14 week time points, which were after the 6-week peak of serum Ab titers (**Figure 2B**). Living mice were bled bi-weekly, and groups of mice were sacrificed at 2, 4, 6, 8, and 14 weeks. At the time of sacrifice, CSF, blood, and brains were harvested. #: signifies a mouse death, as detailed in the **Supplemental Materials**, including one 4-week ventrally-induced mouse, one 8-week dorsally-induced boosted mouse, and two 14-week dorsally-induced unboosted mice. **(C)** Behavioral studies were carried out 1-2 weeks before sacrifice. Serum and CSF NMDAR antibody titer, mouse behavior, hippocampal NMDAR cluster density, and brain immune cell entry and cytokine content were examined.

**Figure 2:**
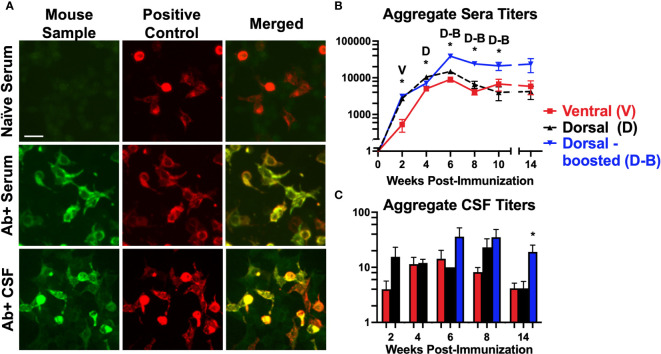
Mice immunized against the GluN1 ATD develop antibodies to NMDARs: **(A)** Representative images of NMDAR (GluN1 subunit)-expressing HEK293 cells incubated with mouse serum or spinal fluid (first column, Mouse Sample) and a commercial antibody to GluN1 (second column, Positive Control), and probed with anti-mouse IgG (green) and anti-rabbit IgG (red). Merged images (3^rd^ column) show co-localization (yellow) between the Abs in the mouse samples and the commercial NMDAR Abs. Scale bar = 10 μm. The sample in the first row (Naïve Serum) was collected prior to immunization from a mouse that later produced Abs, whereas the samples in the second and third rows were NMDAR Ab-positive serum and CSF collected from immunized mice. **(B, C)** The HEK293 cell based assay was used to evaluate for serum NMDAR Abs in mice pre-immunization and at 2-week intervals after immunization. CSF samples were collected just prior to brain harvesting. Titers for each induction group (Ventral (V), Dorsal (D), or Dorsal-boosted (D-B)) are plotted over time for serum **(B)** and at each harvest timepoint for CSF **(C)** as mean, with error bars showing standard error of the mean. The y-axis is displayed in log scale. The numbers of mice per group per time point are detailed in **Supplementary Table 1** (at least 8 per group for serum and at least 2 per group for CSF). Unpaired two-tailed Mann Whitney U tests were used to determine statistically significant differences between the means of the groups for CSF titer time points with 2 groups **(C)** and a Kruskal-Wallis 1-way ANOVA with Dunn’s multiple comparison test was performed to determine differences for time points with 3 groups **(B, C)**. *: *P <= 0.05.* Letters above the stars indicate which induction group statistically differed from the 2 others. At 8 weeks, the mean CSF titer of boosted mice was statistically greater than that of ventrally-induced mice (*P = 0.007*), but not dorsally-induced unboosted mice (*P = 0.5*).

**Figure 3:**
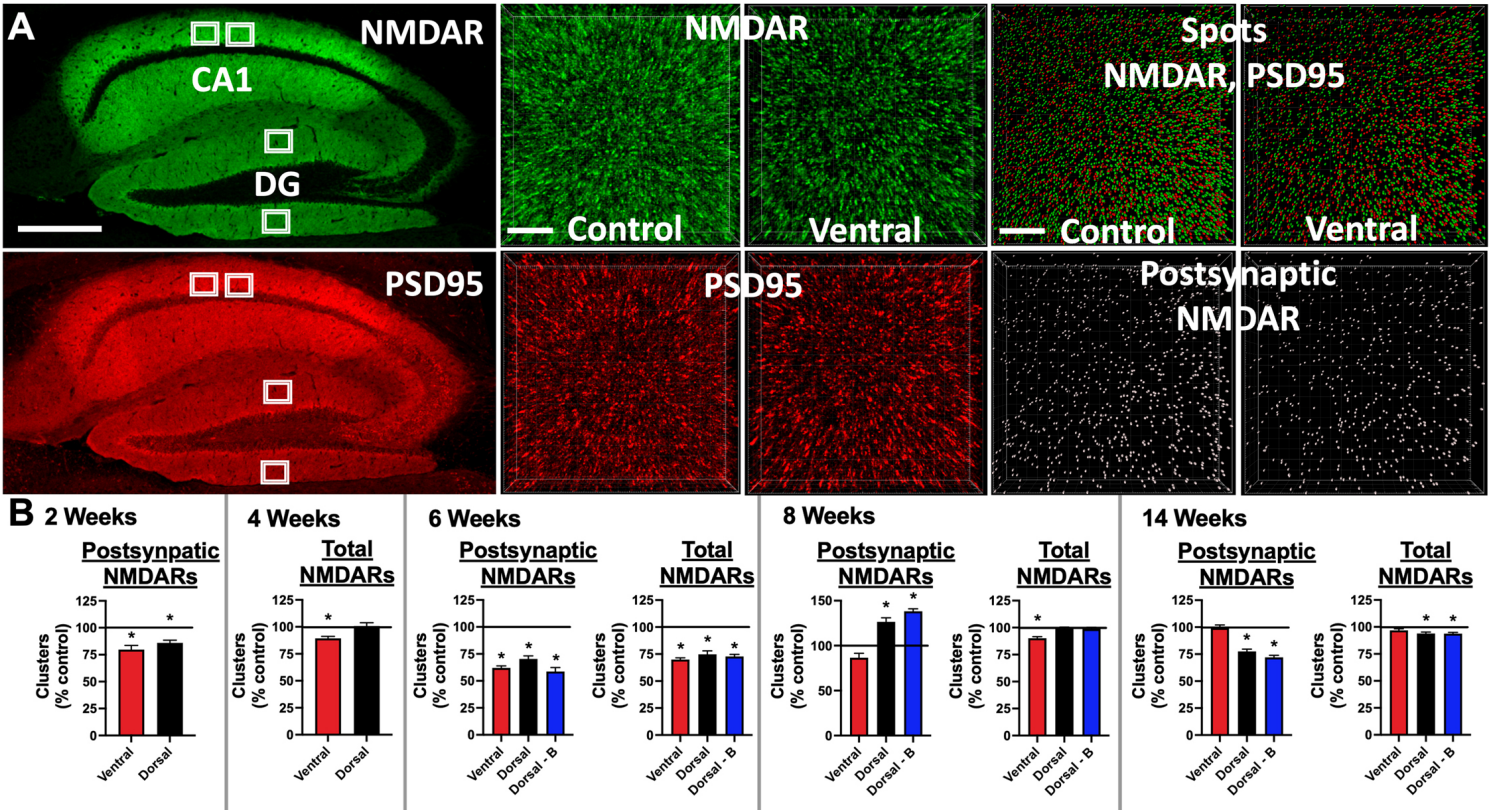
Mice immunized against the GluN1 ATD have decreased hippocampal cell surface and postsynaptic NMDAR cluster density: **(A)** Brains were fixed, cryoprotected, and frozen at 2, 4, 6, 8, and 14 weeks post-immunization. 10 μm sections were stained with human CSF containing NMDAR Abs (green), permeabilized, and then stained with a commercial Ab against PSD95 (red). Left: representative images from a 14 week time point control mouse. Scale bar = 500 μm. Confocal imaging was used to acquire four 82 x 82 x 3 μm^3^ stacks from the CA1 and dentate gyrus (DG) areas (white boxes). 3 smaller 20.5 x 20.5 x 3 μm^3^ stacks were selected for quantification from deconvolved images, amounting to 12 total regions per stained mouse half brain. Middle: representative 6 week images from an unimmunized control mouse (Control) and ventrally-immunized mouse (Ventral). Note the relative decrease in cell surface NMDAR clusters between Control and Ventral. Scale bar = 4 μm. Top right: A spot detection algorithm was used to detect NMDAR and PSD95 clusters (Spots) and co-localized (within 0.2 μm) postsynaptic NMDAR clusters (bottom right, Postsynaptic NMDAR). Representative images from 6 week time point control (Control) and ventrally-immunized mice (Ventral). Note the relative decrease in postsynaptic NMDAR clusters between Control and Ventral. Scale bar = 4 μm. **(B)** Mean postsynaptic NMDAR and total cell surface NMDAR cluster density for each induction group (Ventral, Dorsal, or Dorsal-boosted (Dorsal-B)) as a percentage normalized to the mean of the unimmunized control group, which is represented as a horizontal line at 100%. Error bars show standard errors of the normalized means. There were 2 stained half brains per group for the 2, 4, and 6-week timepoints, 3 stained half brains at 8 weeks, and 5 stained half brains per group at 14 weeks. Dorsally-boosted mice were only represented at 6 weeks and beyond, as boosting occurred at 4 weeks. Results were tested for normality. For normally-distributed data, a 1-way ANOVA with Tukey’s multiple comparison test was performed to determine differences between group means. For non-normally-distributed data, a Kruskal-Wallis 1-way ANOVA with Dunn’s multiple comparison test was performed to determine differences between group means. Graphs with at least one treatment group mean that was statistically different from the mean of the control group (signified by a ‘*’) are displayed. *: *P < 0.05*.

**Figure 4:**
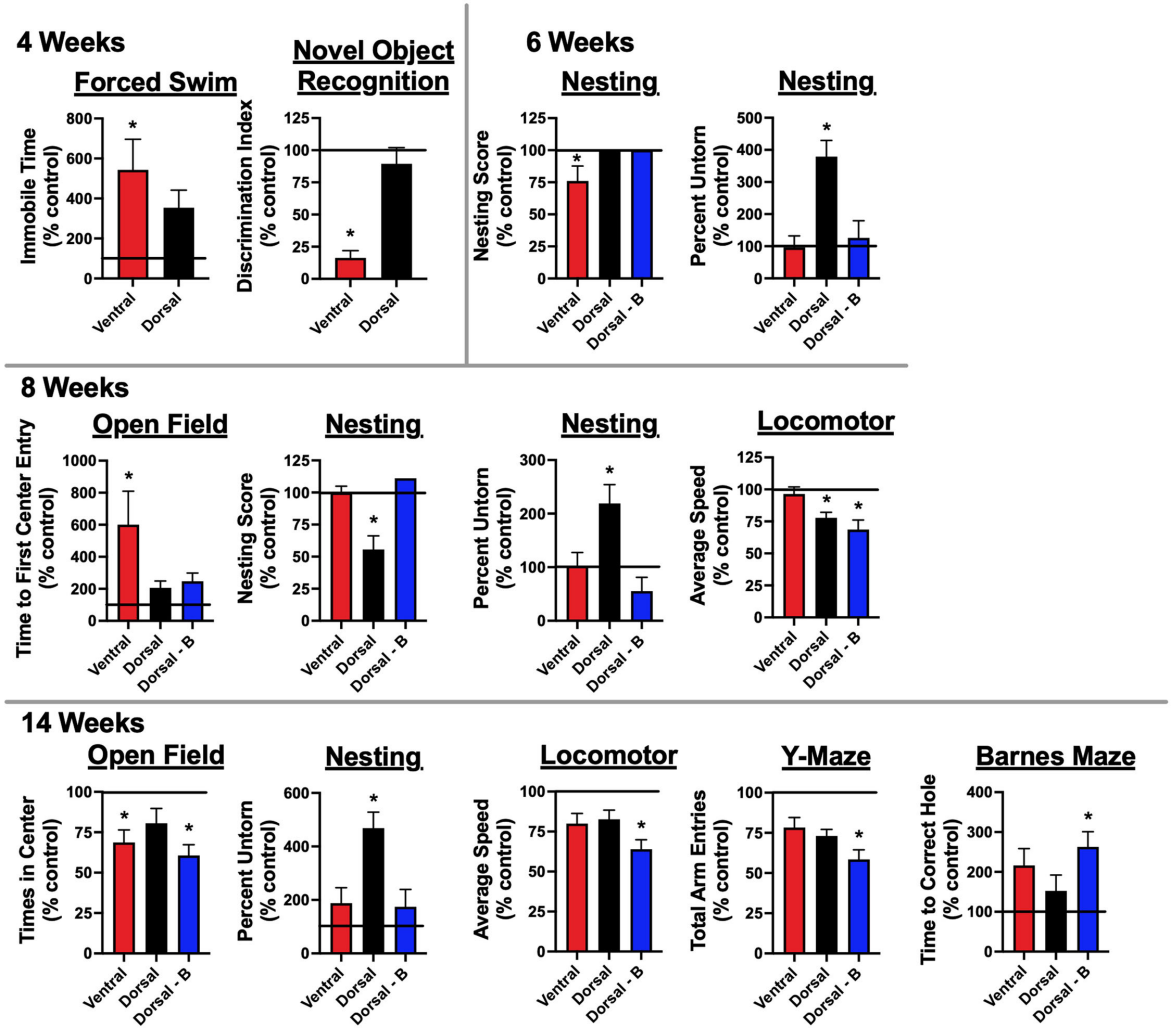
Mice immunized against the GluN1 ATD develop memory deficits and signs of depression and anxiety: One (2, 4, and 6-week timepoints) to two (8 and 14-week timepoints) weeks prior to sacrifice, mice underwent behavioral testing. They were tested for memory deficits via novel object recognition (NOR) testing. Depression was assessed through forced swimming. Anxiety was tested for via open field assessment. General well-being was assessed through nest building. Motor and general function were assessed through locomotor monitoring. Additional memory tests, Y-maze and Barnes maze, were carried out for the 8 and 14 week cohorts, which were tested after the 6-week peak of serum Ab titers (**Figure 2B**). There were 5 mice tested per group (Control, Ventral, Dorsal, and Dorsal-boosted (Dorsal-B)) at the 2, 4, and 6 week time points, except for 4 mice at the 4 week time point in the ventrally-induced group, as one mouse in this group died during intravenous injection of pertussis toxin. Its replacement mouse was from a different litter and was housed with fewer litter mates. It was thus excluded from behavioral analyses. Boosted mice only had testing from 6 weeks and beyond, as boosting occurred at 4 weeks. There were 10 mice tested per group at the 8 and 14 week time points, with the exception of 9 dorsally-boosted mice at 8 weeks and 8 dorsally-induced unboosted mice at 14 weeks, due to mouse deaths in those groups. For the Barnes maze analysis, mice that demonstrated boredom with the task and did not attempt to look for the escape hole during the probe trial were excluded. This included 2 control mice at the 14 week time point. Results were tested for normality. For normally-distributed data, a 1-way ANOVA with Tukey’s multiple comparison test was performed to determine differences between group means. For non-normally-distributed data, a Kruskal-Wallis 1-way ANOVA with Dunn’s multiple comparison test was performed to determine differences between group means. Behavioral tests with at least one treatment group mean that was statistically different from the mean of the control group (signified by a ‘*’) are displayed, with data shown as a mean percentage normalized to the mean of the unimmunized control group, which is represented as a horizontal line at 100%. Error bars depict the standard error of the normalized mean. *: *P < 0.05*.

**Figure 5:**
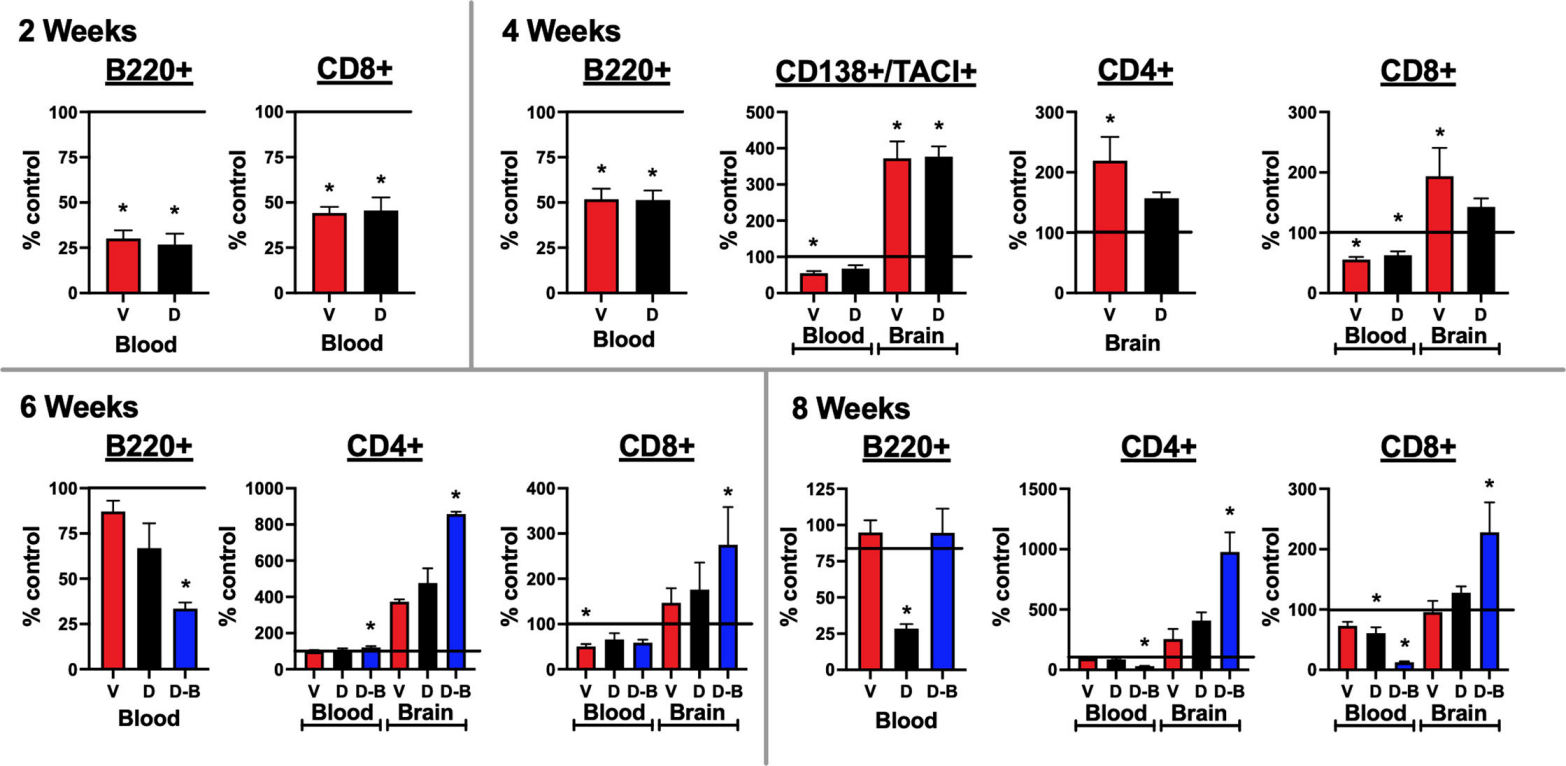
Lymphocytes enter the brains of mice immunized against the GluN1 ATD: Blood and half brain samples were collected at the time of sacrifice at 2, 4, 6, and 8 weeks. At the 2 and 4-week time points, a single additional naïve age and sex-matched control mouse was added, providing two additional half-brains. At the 6-week timepoint, two additional naïve age and sex-matched control mice were added, providing four additional half-brains. A single spleen at each timepoint was also processed and stained for lymphocytes, as a positive staining control. Unstained cells were used as a negative staining control. Cells were stained for CD4+ and CD8+ T cells (CD45+/CD3+/B220-), B220+ B cells (CD45+/CD3-/B220+), and CD138+/TACI+ plasma cells (CD45+/CD138+/TACI+). Flow cytometry results were quantified as a percentage of live cells. They are expressed as the treatment group mean percentage normalized to the mean of the unimmunized control group, which is represented as a horizontal line at 100%. Error bars show standard errors of the normalized means. At the 2 and 4 week time points, for blood, there were 3 samples from each immunized group and 4 samples from the control group, and for brain, there were 3 half brains from each immunized group and 5 half brains from the control group. At the 6 week time point, for blood, there were 3 samples from each immunized group and 5 samples from the control group and for brain, there were 3 half brains from each immunized group and 7 half brains from the control group. At the 8 week time point, there were 7 blood samples and half brains for the Control, Ventral (V), and Dorsal (D) unboosted mice and 6 blood samples and half brains for the Dorsal-boosted (D-B) mice, due to the death of a mouse in this group. The recording run malfunctioned for one of the 8 week control brain samples, which was excluded from the analyses. Dorsally-boosted mice were only represented at 6 and 8 weeks, as boosting occurred at 4 weeks. Results were tested for normality. For normally-distributed data, a 1-way ANOVA with Tukey’s multiple comparison test was performed to determine differences between group means. For non-normally-distributed data, a Kruskal-Wallis 1-way ANOVA with Dunn’s multiple comparison test was performed to determine differences between group means. Graphs with at least one treatment group mean that was statistically different from the mean of the control group (signified by a ‘*’) are displayed. *: *P < 0.05.*.

**Figure 6:**
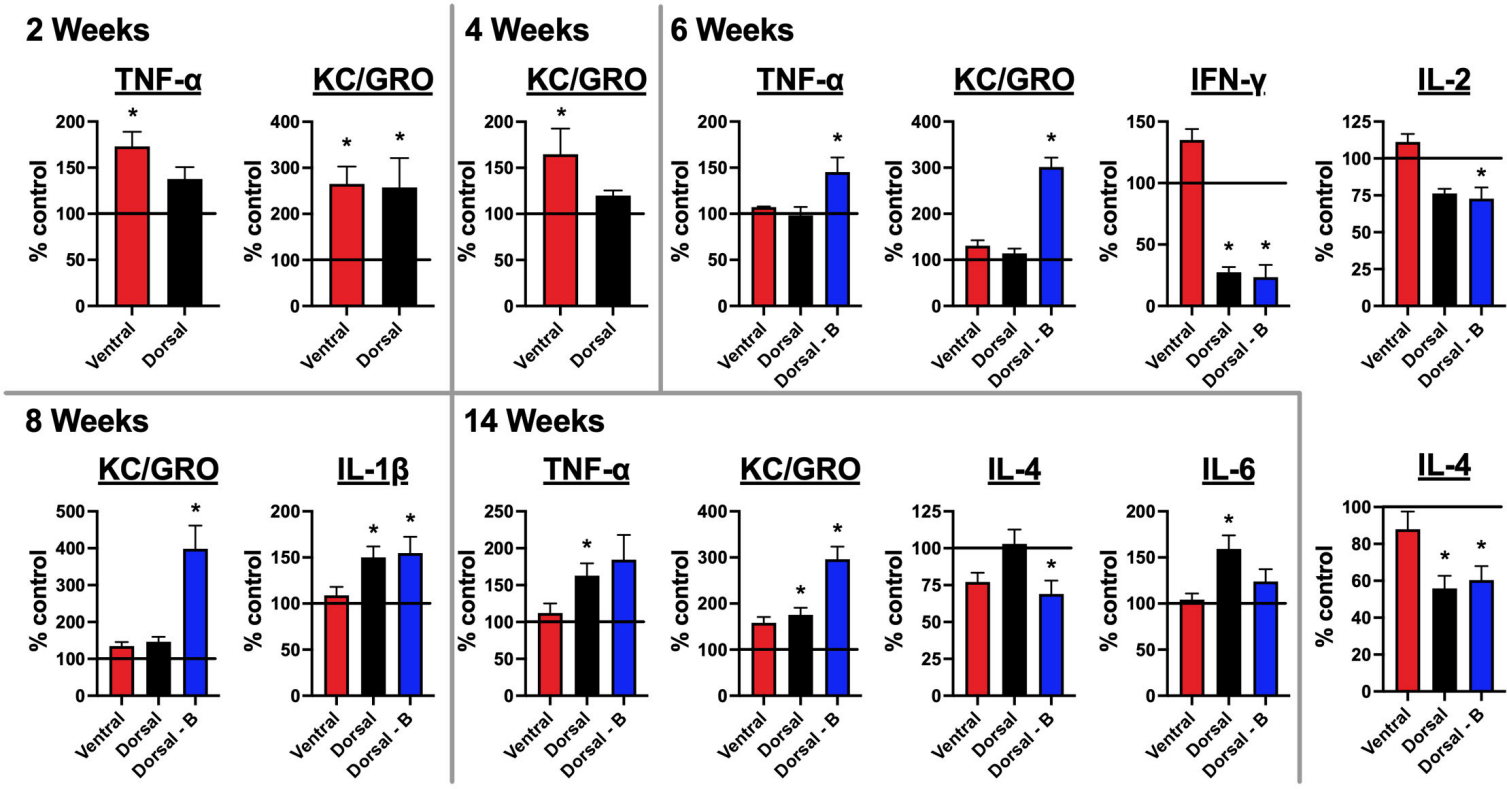
Mice immunized against the GluN1 ATD have altered brain cytokine profiles: A pro-inflammatory kit was used to evaluate cytokine levels for IFN-γ, IL-1β, IL-2, IL-4, IL-5, IL-6, IL-10, IL-12p70, KC/GRO (CXCL1), and TNF-α. Half brains were homogenized and spun down. Supernatants were tested for cytokines. Brains at 4 and 8 week time points were collected prior to boosting. Cytokine levels were quantified as pg per g whole brain tissue and are expressed as the treatment group mean percentage normalized to the mean of the unimmunized control group, which is represented as a horizontal line at 100%. Error bars show standard errors of the normalized means. There were 5 mice per group (Control, Ventral, Dorsal, and Dorsal-boosted (Dorsal-B)) at the 2, 4, and 6-week time points. Boosted mice only had samples from 6 weeks and beyond. There were 10 mice per group at the 8 and 14 week time points, with the exception of 9 dorsally-boosted mice at 8 weeks and 8 dorsally-induced mice at 14 weeks, due to mouse deaths in those groups. One ventrally-induced mouse in the 14 week group was excluded as an outlier (as confirmed by Prism software) due to values that were many magnitudes different from all other mice. Results were tested for normality. For normally-distributed data, a 1-way ANOVA with Tukey’s multiple comparison test was performed to determine differences between group means. For non-normally-distributed data, a Kruskal-Wallis 1-way ANOVA with Dunn’s multiple comparison test was performed to determine differences between group means. Graphs with at least one treatment group mean that was statistically different from the mean of the control group (signified by a ‘*’) are displayed. *: *P < 0.05*.

The current study was undertaken to determine the optimal approach and timing for active immunization against the ATD of the GluN1 in a rodent model of NMDARE through head-to-head comparisons of groups of immunized mice. As the field is still at an early stage in developing animal models of AE/NMDARE, it is conceivable that variations in induction methodology could result in important differences in the resultant model. To limit permutations, and given the reported inconsistencies in results between the GluN1_359-378_ peptide used in one study (12) and the GluN1_359-377_ peptide used in the other (13), we used the 30 amino acid length peptide described in the latter (13) in all immunized groups, also opting for their repeated immunization approach (at 0, 4, and 8 weeks) via the dorsal surface as one of our main groups (=Dorsal-boosted, **Figures 1A, B**). Our other treatment groups were 1) mice that were induced via the dorsal surface but not boosted (=Dorsal), encompassing the method reported by Wagnon et al. (12), and 2) unboosted mice that were immunized via the ventral surface (=Ventral), using the method our laboratorfy typically uses to induce EAE (18). Except for the 12-week time-point (due to the timing of behavioral experiments), mice were sampled every 2 weeks for NMDAR Abs in serum (**Figure 2B**). Behavioral tests, as outlined in **Figure 1C**, were carried out 1-2 weeks prior to harvest. Spinal fluid and brains were harvested at 2, 4, 6, 8, and 14 weeks post immunization to examine for CSF NMDAR Abs (**Figure 2C**). The biochemical (**Figure 3**), behavioral (**Figure 4**) and immunologic (**Figures 5**, **6**) effects of the Abs were summarized. The overall experimental scheme is summarized in **Figures 1A–C**.

## Methods

Please refer to the **Supplementary Materials** for more detailed descriptions of the methodology.

### Immunization with GluN1 ATD peptide

8-week-old female C57BL/6 mice (Jackson Laboratories, Bar Harbor, ME; 5 per group for 2, 4, and 6-week timepoints and 10 per group for 8 and 14-week timepoints) were anesthetized with 3% isoflurane. Boosted dorsally-induced mice were immunized as per Ding et al. (13), summarized in the **Supplementary Materials**. Unboosted dorsally-induced mice were injected once intraperitoneally with 400 ng of pertussis toxin at the time of immunization and did not receive booster injections. Ventrally-induced mice (15, 18) were immunized via four 25 μL subcutaneous injections of the emulsion into the bilateral axillary and inguinal regions with a peptide emulsion prepared with CFA supplemented with 16 mg/mL killed and dessicated *Mycobacterium tuberculosis (Mtb)*. Mice were then injected intravenously with 250 ng pertussis toxin via a tail vein at the time of immunization, as well as 48 hours later. Controls were age and sex-matched naïve mice.

### Serum collection

Mice were anesthetized with 3% isoflurane and retro-orbital bleeds were performed using heparinized glass hematocrit capillary tubes (BD) pre-immunization and every 2 weeks after immunization, except for week 6 for the 8-week mice and week 12 week for the 14-week mice, when they were undergoing behavior testing. Blood samples were collected at 4 and 8 weeks before mice were boosted. Clotted blood was spun for 10 minutes at 1200g and 4°C and supernatant (serum) was collected.

### CSF collection

CSF was collected (20) just before harvesting the brain. At 2, 4, 6, 8, and 14 weeks post immunization, mice were anesthetized with 3% isoflurane and placed prone into a stereotactic holder (Harvard Apparatus, Holliston, MA), with their snout angled downwards. A skin incision was made inferior to the occiput. The subcutaneous tissue and muscles were gently separated from the midline to reveal the dura mater of the cisterna magna, which was punctured with a beveled glass capillary tube (outer diameter 1 mm, inner diameter 0.75 mm, tip 0.5 mm; Clunbury Scientific, Bloomfield Hills, MI) secured in a holder (World Precision Instruments, Sarasota, FL) and mounted to a stereotactic arm. Any CSF contaminated with blood was excluded from analysis.

### Brain collection and processing

After CSF collection, the right renal vein was cut, and anesthetized mice were perfused with 20 mL of ice-cold PBS via cardiac puncture to remove intravascular blood. Brains were collected and split at the midline. A half brain was put into either 10 mL of ice-cold PBS or 5 mL of 4% paraformaldehyde (Thermo Scientific, Waltham, MA) on ice for an hour, before being transferred into 10 mL 40% sucrose in PBS at 4°C for 48 hours (6), then embedded in optimized cutting temperature medium (OCT, Sakura Finetek, Torrance, CA), frozen with dry ice-chilled isopentane, and stored at -80°C. The other half brain was placed into a tube and snap frozen with dry ice-chilled isopentane and stored at -80°C.

### Immunofluorescent cell based assay (CBA) for NMDAR antibody detection

The presence of NMDAR antibodies in mouse sera and CSF was determined using a commercial NMDAR transfected HEK293 CBA kit (Euroimmun, Lübeck, Germany), with slight modifications to the manufacturer’s protocol, as outlined in the **Supplementary Materials** in more detail.

### Behavioral testing

Tasks were chosen to test memory (novel object recognition, Barnes maze, Y maze), learning (fear conditioning), anxiety (open field, O-maze), depressive-like behaviors (forced swim, tail suspension), locomotor activity (horizontal assessment), and general well-being (nesting). Testing protocols are detailed in the **Supplementary Materials**.

### Immunofluorescence and confocal microscopy for NMDAR cluster density analysis

Frozen sucrose-protected half brains (2 per group at the 2, 4, and 6 week time points, 3 per group for the 8 week time point, and 5 per group for the 14 week time point) were sagittally sectioned, stained for NMDAR, permeabilized, stained for PSD95, and imaged via confocal. Clusters were quantified as previously described (7) and summarized in the **Supplementary Materials**.

### Flow cytometry

Infiltrating immune cells were isolated from half brains and blood, stained with anti-mouse antibodies (BioLegend) against CD11b, CD45, CD3, CD4, CD8a, B220, TACI, and CD138, and quantified as per the **Supplemental Materials**. The number of mouse brains and bleeds per treatment group and timepoint are detailed within the legend for **Figure 5**.

### Cytokine analyses

A snap frozen half brain was thawed and cut 4-ways in RIPA lysis buffer (1 mL/150 mg tissue, Millipore, Burlington, MA, USA) supplemented with cOmplete™ protease inhibitor tablets (Roche, Basel, Switzerland) on ice. The tissue was sonicated for 20 seconds (Sonic Dismembrator Model 100, Fisher Scientific, Hampton, NH, USA) and was spun for 30 minutes at 55,000 g at 4°C. The supernatant was collected and stored at -80°C. Cytokine levels were determined by a mouse V-PLEX Proinflammatory Panel 1 kit with capture antibodies to IFN-γ, IL-1β, IL-2, IL-4, IL-5, IL-6, IL-10, IL-12p70, KC/GRO (CXCL1), and TNF-α (Meso Scale Discovery (MSD), Rockville, MD, USA) according to the manufacturer’s protocol, summarized in the **Supplementary Materials**.

### Statistics

Statistical analyses were carried out using GraphPad Prism Software (Version 9.0, Dotmatics, San Diego, CA). Results were expressed as mean with standard error of the mean error bars. Some means were expressed as a mean of each treatment group value normalized to the mean of the control group, expressed as a percentage, with the control group mean = 100%. Groups were tested for normality using D’Agostino, Anderson-Darling, Shapiro-Wilk, and Kolmogorov-Smirnov tests. The means of two different treatment groups were compared using unpaired two-tailed Mann Whitney U tests. Three or greater normally distributed treatment groups were tested with one-way ANOVA analyses followed by Tukey’s multiple comparison tests to determine the differences between treatment means. Three or greater non-normally distributed treatment groups, which failed the four normality tests above, were tested with nonparametric Kruskal-Wallis one-way ANOVA analyses followed by Dunn’s multiple comparison tests to determine the differences between treatment means. A *P*-value of less than or equal to 0.05 was considered statistically significant. Sample size and power calculations are detailed in the **Supplementary Materials**.

### Data availability

Data will be shared upon request from any qualified investigator.

## Results

### Mice injected subcutaneously with GluN1 ATD peptide and immune adjuvants produce NMDAR antibodies in their blood and spinal fluid

Serum was serially collected from all living mice at 2-week intervals, except for mice that were undergoing behavioral testing, and CSF was collected at 2, 4, 6, 8, and 14-week time points, just prior to brain harvesting. A commercial GluN1-transfected HEK293 cell-based assay (**Figure 2A**) was used to determine Ab titers. Ventrally-induced mice initially lagged behind dorsally-induced mice in serum (**Figure 2B**) and CSF (**Figure 2C**) Ab titers, but by 4 weeks, ventrally-induced and dorsally-induced/pertussis naïve mice had similar levels of Abs in their blood and all groups had similar titers in their spinal fluid. Starting at 6 weeks, the boosted group serum and CSF Ab titers were above those of unboosted mice at all subsequent time points studied. In all groups, serum Ab titers peaked at 6 weeks. CSF Ab titers were highest at 6 weeks in the ventrally and dorsally-induced boosted mice and at 8 weeks in the dorsally-induced unboosted mice. 14 weeks after immunization, CSF Ab titers were significantly higher in boosted versus unboosted mice.

### Mice with NMDAR Abs have decreased hippocampal cell surface and postsynaptic NMDARs

Brain samples were collected at 2, 4, 6, 8, and 14 weeks after immunization, sagittally sectioned and stained for NMDARs and PSD95 (**Figure 3A**). Non-permeabilized tissue was stained for NMDARs, capturing total cell surface NMDAR clusters. PSD95 staining was performed after tissue permeabilization. The densities of the cell surface (**Figure 3A**, green spots) and postsynaptic (**Figure 3A**, colocalized NMDAR+/PSD95+) NMDAR clusters were quantified (**Figure 3B**) from 3 subsections within two CA1 and dentate gyrus (DG) hippocampal regions (**Figure 3A**, white boxes). At the 6-week timepoint, the time of peak serum NMDAR Ab production in all groups, the mean NMDAR cluster density (**Figure 3B**) was significantly lower in all immunized groups compared to control mice. The mean hippocampal NMDAR cluster density, whether total cell surface or postsynaptic, was lower in the ventrally-immunized group compared to naïve control mice at the 2, 4, 6, and 8 week time points, in all regions studied, with the exception of the DG at 4 weeks. For postsynaptic NMDAR cluster density (**Figure 3B**), all statistically significant differences between the control and ventrally-induced means occurred before 8 weeks. Total cell surface NMDAR cluster density in the ventral group (**Figure 3B**) was statistically lower than all other groups at 4 and 8 weeks and control mice at 6 weeks. By 14 weeks, the mean NMDAR cluster density was not different between ventrally-induced and non-immunized mice (**Figure 3B**). Differences in NMDAR cluster density compared to control mice were more variable for dorsally-immunized mice, which showed a mild paradoxical increase in postsynaptic NMDAR cluster density compared to naïve mice at 8 weeks. At 14 weeks, mean NMDAR cluster density was significantly lower in dorsally-induced mice compared to controls, with the means slightly lower in boosted compared to unboosted mice (**Figure 3B**). Graphs where the treatment group NMDAR cluster results were not statistically significant from naïve mice are shown in **Supplementary Figure 1**. PSD95 cluster density means were also quantified (**Supplementary Figure 2**).

### Mice with NMDAR Abs demonstrate deficits in memory, anxiety, and depressive behavior

In the 1-2 weeks prior to sacrifice, mice were tested for memory deficits (novel object recognition (NOR)), anxiety (open field), and depression (forced swimming). Motor function (locomotion) and general well-being (nesting) were also tested. Additional tests for memory (Barnes maze, Y-maze, fear conditioning), anxiety (O-maze), and depression (tail suspension) were added at the 8 and/or 14-week time points (**Figure 1C**), given that the peak serum antibody titers occurred at 6 weeks (**Figure 2B**). No significant differences from control mice were noted for fear conditioning, O-maze, or tail suspension tests. Significant behavioral disruptions were not noted until 4 weeks after immunization (**Figure 4**), at which point the ventrally-induced mice demonstrated increased immobile time on forced swim testing compared to naïve control mice and decreased preference for the novel object in NOR testing compared to dorsally-immunized and naïve control mice. On a test of anxiety at the 4-week timepoint, ventrally-immunized mice also spent significantly less time in the center of an open field than dorsally-immunized mice, but the difference from control mice was not statistically significant (**Supplementary Figure 3A**). Behavioral differences noted in ventrally-induced mice after 4 weeks included nesting scores, which were significantly lower than all other groups at 6 weeks, and measures of anxiety, as mice took longer to enter the center of an open field than non-induced control mice at 8 weeks and they entered the center of the open field fewer times than naïve mice at the 14 week time point.

Dorsally-induced unboosted mice did not show significant behavioral differences from control mice, aside from in nest building and locomotion. In the nest building test, they made poorly-formed nests (8 weeks) and left large chunks of nesting material unshredded (6, 8, and 14 weeks). This became evident at 6 weeks and later, when the protocol was changed to introduce socially-housed mice to the nesting material a few days before being singly housed for the nesting testing. Their nesting measures were significantly different from all other groups in the graphs in **Figure 4**, aside from the ventrally-induced mice at 14 weeks (*P=0.07*). In locomotor testing at 8 weeks, dorsally-immunized mice (unboosted and boosted) spent less time exploring than control mice in an open field paradigm, at times preferring to be stationary, and preoccupied with repetitive self-grooming behaviors. This amotivational behavior persisted in boosted mice at the 14 week time point and was also reflected in a lower number of total arm entries into the Y-maze by boosted mice at 14 weeks as compared to non-immunized mice, regardless of whether the retention interval was 30 minutes or 2.5 hours (**Supplementary Figure 3B**). For boosted mice, the greatest number of statistically significant behavioral differences from controls were exhibited at the 14-week timepoint. In addition to the aforementioned differences in locomotion and Y-maze behavior, at 14 weeks post-immunization, boosted mice also entered the center of the open field fewer times and took longer to find the correct hole in the Barnes maze probe trial, as compared to naïve mice. Graphs outlining mean treatment group behavior results that were not statistically significant from naïve mice are shown in **Supplementary Figures 4A–E**.

### Lymphocytes enter the brains of mice immunized against NMDARs

Brain samples were collected at 2, 4, 6, and 8 weeks post immunization. The blood and brain samples were processed for flow cytometry and stained for lymphocytes, including B cells, plasma cells, and T cells (CD4+ and CD8+, **Figure 5**). The gating paradigms are shown in **Supplementary Figures 5A–D**. Significant differences from control mice in B220+ B cells were only detected in blood. As compared to non-immunized mice, B cells were significantly decreased in immunized mice at 2 and 4 weeks. At 6 weeks, they were only significantly decreased in boosted mice. At 8 weeks, they were significantly decreased in dorsally-induced non-boosted mice, versus all other groups. Activated B cells in the plasma cell lineage (CD138+/TACI+) were only detected at the 4 week time point. In the blood, they were significantly decreased as compared to naïve control mice in only ventrally-induced mice. In the brain, they were significantly increased in immunized mice (ventrally and dorsally-induced) compared to control mice. In the blood, at 6 weeks there were significantly more CD4+ T cells, and at 8 weeks there were significantly fewer CD4+ T cells in boosted mice only compared to control mice. In the brain, at 4 weeks, there were significantly more CD4+ T cells in only the ventrally-induced mice, whereas at 6 weeks, there were significantly more CD4+ T cells in only the boosted mice compared to naïve mice. At 8 weeks, there were significantly more CD4+ T cells in the boosted mice than in all other groups. In general, CD8+ T cells decreased in the blood of immunized versus control mice, although at 6 weeks there were significantly fewer cells in only the ventrally-induced group, and at 8 weeks there were significantly fewer cells in only the dorsally-immunized mice (unboosted and boosted). In the brain, at 4 weeks there were significantly more CD8+ T cells in only the ventrally-induced mice, and at 6 and 8 weeks there were significantly more CD8+ T cells in only the boosted mice, compared to naïve mice. Graphs where blood and brain lymphocyte results were not statistically significant from naïve mice are shown in **Supplementary Figure 6**.

### Brain cytokine levels are altered in mice immunized against NMDARs

Brain samples collected at 2, 4, 6, 8, and 14 weeks after immunization were tested for the presence of 10 cytokines with a commercial pro-inflammatory kit: IFN-γ, IL-1β, IL-2, IL-4, IL-5, IL-6, IL-10, IL-12p70, KC/GRO (CXCL1), and TNF-α (**Figure 6**). Two weeks after immunization, two cytokines were significantly increased in immunized over naïve controls – KC/GRO (in both dorsally and ventrally-induced mice) and TNF-α (in only ventrally-immunized mice). By 4 weeks post-immunization, KC/GRO remained significantly elevated over control mice only in the brains of ventrally-induced mice. By 6 weeks post-immunization, which corresponded with peak serum NMDAR Ab titers in all groups (**Figure 2B**), multiple cytokines were significantly different from controls, but only in dorsally-induced mice. The 6-week time point was 2 weeks after boosting. At 6 weeks, whereas IFN-γ and IL-4 were significantly decreased in unboosted and boosted dorsally-induced mice compared to controls and ventrally induced mice (IFN-γ), KC/GRO and TNF-α were significantly increased and IL-2 was significantly decreased only in boosted mice. Boosted mice were greater than all other groups for KC/GRO, greater than unboosted dorsal and control mice for TNF-α, and less than ventral and control mice for IL-2. By 8 weeks post-immunization, prior to re-boosting, as compared to non-immunized controls, IL-1β was increased in dorsally-induced mice (non-boosted and boosted), whereas KC/GRO remained significantly increased in boosted over all other groups of mice. In the 14 weeks after immunization group, KC/GRO remained elevated in boosted mice over all other groups and it was increased in unboosted dorsal mice over naïve mice. IL-6 and TNF-α were elevated only in dorsally unboosted mice over control (IL-6 and TNF-α) and ventrally-induced mice (IL-6). IL-4 was decreased only in boosted mice over naïve and dorsal unboosted mice. Results for the cytokines IL12p70 and IL-10 were not above the threshold of detection of the assay at any time point (**Supplementary Table 2**). Graphs showing brain cytokine results that were not statistically significant from naïve mice are shown in **Supplementary Figures 7A–E**.

## Discussion

This study compared 3 different approaches of active immunization against the N-terminal domain of the NMDAR GluN1 subunit, chosen to 1) replicate Ding et al. (13)’s conditions (Dorsal-boosted), 2) encompass Wagnon et al. (12)’s conditions (Dorsal), and 3) utilize an EAE-based methodology used in our laboratory (15–18) (Ventral). While antibody titers (**Figure 2**) initially lagged in ventrally-induced mice, by 4 weeks post-immunization, this group had similar serum NMDAR Ab titers to dorsally-immunized mice. Serum antibody titers in all groups peaked at 6 weeks, while CSF titers peaked at 6 weeks in the ventrally-immunized and dorsal boosted group and at 8 weeks in the dorsal unboosted group. The CSF antibody titers were consistent with the diffusion of immunoglobulin from the periphery (21), as opposed to intrathecal antibody synthesis.

At 6 weeks post-immunization, at the peak of serum NMDAR antibody titers, all immunized groups had reduced densities of postsynaptic and total hippocampal NMDAR clusters as compared to non-immunized control mice. NMDAR cluster density increased in dorsally-induced mice (unboosted and boosted) at 8 weeks, potentially indicating receptor upregulation, possibly related to concomitant increases in the cytokine IL-1β (**Figure 6**), which has been implicated in learning and memory (22), in addition to inflammation and seizures (23). Slight coinciding increases in hippocampal PSD95 cluster density in dorsally-induced mice at this time point (**Supplementary Figure 2**) suggest that immunization may have triggered synaptic remodeling, but future studies with larger numbers of mice are needed to more carefully examine the effects of immunization on hippocampal synaptic structure. Behaviorally, depressive behavior and memory deficits were evident at the 4 week timepoint in ventrally-induced mice, and signs of anxiety were seen in these mice from 4 out to 14 weeks post-immunization (**Figure 3**; **Supplementary Figure 1A**). Repeated boosting was necessary to produce broad behavioral changes at 14 weeks. In ventrally-immunized mice, hippocampal NMDAR clusters generally decreased from 2 to 8 weeks post-immunization but were normalized by the 14 week time point (**Figure 4**).

The profiling of adaptive immune cells (**Figure 5**) demonstrated an overall trend in immunized mice, except for CD4+ T cells, as lymphocytes generally decreased in the blood and increased in the brain compared to naïve control mice. This may reflect the shuttling of immune cells from the periphery to the CNS. Immunization appeared to promote the maturation of B cells into plasma cells, which then proceeded from the bloodstream into the brain by the 4 week timepoint. T cells were seen in the brain 4 weeks post-immunization in ventrally-immunized mice. However, for dorsally-induced groups, statistically significant increases in brain T cells were only apparent after boosting, starting 6 weeks after immunization.

Immunization also provoked changes in brain cytokines (**Figure 6**). Even though the earliest changes were evident at 2 weeks, most changes in cytokines occurred in dorsally-induced mice at 6 weeks post-immunization, when serum titers of NMDAR Abs were the highest. As compared to unboosted and control mice, reimmunization altered the cytokine profile in boosted mice, raising TNF-α at 6 weeks, KC/GRO at 6 and 8 weeks, decreasing IL-2 levels at 6 weeks, and decreasing IL-4 levels 14 weeks post-immunization. The overall effects of immunization in the different induction groups are summarized in **Table 1**.

**Table 1:** Summary of immunization effects over time by induction group: Outcomes of immunization (antibody titers, mouse behavior, NMDAR cluster quantification, immune cell presence in blood and/or brain, and brain cytokines) are summarized for the 2, 4, 6, 8, and 14 week time points, organized by induction method – Ventral, Dorsal, and Dorsal-boosted.

		*Time Points (weeks)*				
		2	4	6	8	14
**Ventral**	**Ab Titers**	serum (+) & CSF (+)	serum (++) & CSF (++)	serum (++)* & CSF (++)*	serum (++) & CSF (+)	serum (++) & CSF (+)
	**Behavior**		↓ memory & ↑ depressive	poor nesting	↑ anxiety	↑ anxiety
	**NMDAR** **Clusters**	↓ synaptic	↓ cell surface	↓ synaptic & cell surface	↓ cell surface	
	**Immune** **Cells**	**Blood:** ↓ B220 & CD8	**Blood:** ↓ B220, CD8, & PCs **Brain:** ↑ PCs, CD4 & CD8	**Blood:** ↓ CD8		
	**Cytokines**	↑ TNF-α & KC/GRO	↑ KC/GRO			
**Dorsal**	**Ab Titers**	serum (++) & CSF (++)	serum (+++) & CSF (++)	serum (+++)* & CSF (++)	serum (++) & CSF (+++)*	serum (++) & CSF (+)
	**Behavior**			poor nesting	poor nesting & locomotion	poor nesting
	**NMDAR** **Clusters**	↓ synaptic		↓ synaptic & cell surface	↑ synaptic	↓ synaptic & cell surface
	**Immune** **Cells**	**Blood:** ↓ B220 & CD8	**Blood:** ↓ B220 & CD8 **Brain:** ↑ PCs		**Blood:** ↓ B202 & CD8	
	**Cytokines**	↑ KC/GRO		↓ IFN-γ, IL-4	↑ IL-1β	↑ TNF-α & ↑ IL-6
**Dorsal - boosted**	**Ab Titers**	serum (++)	serum (++)	serum (+++)* & CSF (+++)*	serum (+++) & CSF (+++)	serum (+++) & CSF (++)
	**Behavior**				poor locomotion	↑ anxiety; poor locomotion/motivation; ↓ memory
	**NMDAR Clusters**			↓ synaptic & cell surface	↑ synaptic	↓ synaptic & cell surface
	**Immune** **Cells**			**Blood:** ↓ B220 & ↑ CD4 **Brain:** ↑ CD4 & CD8	**Blood:** ↓ CD4 & CD8 **Brain:** ↑ CD4 & CD8	
	**Cytokines**			↑ TNF-α, KC/GRO & ↓ IFN-γ, IL-2 & IL-4	↑ KC/GRO & IL-1β	↑ KC/GRO & ↓IL-4

Black squares signify either no statistically significant change from control or else not assessed. Yellow squares signify time points where the most changes appear for each treatment group, according to the different outcomes measured. Serum titers: + <= 999, ++ = 1000 – 9,999, +++ >= 10,000; CSF titers: + <= 9, ++ = 10 – 19, +++ >= 20. * = peak serum or CSF titer. Abbreviations: Ab, antibody; CD, cluster of differentiation; CSF, cerebrospinal fluid; IFN, interferon; IL, interleukin; KC/GRO, keratinocyte chemoattractant/human growth-regulated oncogene; NMDAR, anti-N-methyl-D-aspartate receptor; PCs, plasma cells; TNF, tumor necrosis factor.

Our work compared existing active immunization models (12, 13) to a new NMDARE model in which mice were immunized on their ventral surface, as is done in our laboratory protocol for EAE (15). In aggregate, when incorporating all outcome measures surveyed, changes appeared earlier in ventrally compared to dorsally-induced mice (**Table 1**). Although all methods resulted in the robust production of antibodies against NMDARs in the blood and the CSF of immunized mice (**Figure 2**) and the reduction of post-synaptic and total hippocampal NMDAR clusters at 6-weeks post-immunization, the time of the peak of antibody production (**Figure 4**), only the ventral immunization method resulted in a translationally-relevant phenotype (1, 6) at the 4 week time point. The ventrally-induced group is also the only group where T-cells entered the brain and cerebral cytokine changes were evident 4 weeks post-immunization. In many NMDARE patients, we do not know the time course between their priming against NMDARs and their manifestation of symptoms. The exception is post-herpetic encephalitis NMDARE, where patients typically develop symptoms about 2-4 weeks after a herpes infection of the brain (24, 25). Thus, the time course of changes in the ventrally-induced mice matches well with that in post-herpetic NMDARE, supporting the strong translational potential of this previously unpublished model of active immunization against NMDARs.

A similar NMDARE-like phenotype was not seen again until 14 weeks post-immunization in the dorsally boosted mice, demonstrating that multiple rounds of boosting were required to achieve this phenotype in dorsally-immunized mice. Boosting also led to higher titers of NMDAR Abs overall and to the entry of T cells into the brains of dorsally-induced mice. Interestingly, boosting did not lead to an increase in cerebral plasma cells or other B cells, as was noted in immunized mice at the 4 week time point, suggesting that the antibodies may be generated peripherally, likely from memory cells that are reactivated by the new antigen challenge (boosting). When we ventrally induce EAE, we do not reimmunize mice, as we typically see effects within a few weeks. Thus, in the current work, where we did not know the results *a priori*, we did not have a boosted ventral group of mice. Across most outcome measures, changes in ventrally-induced mice diminished over time. Future work could compare boosted ventrally- and dorsally- induced groups to see if boosting ventrally-immunized mice helps to maintain their clinically-relevant phenotype over time. It is unknown why significant differences were seen between ventral and dorsal immunization approaches, although subcutaneous injection into the axillae and groin places the inflammatory peptide/adjuvant emulsion in close proximity to regional lymph nodes, at least half of which (axillary) are closer to the brain and its draining cervical lymph nodes than those draining the mouse tail base. Additionally, our ventral approach utilizes higher *Mtb* and pertussis toxin concentrations than those used by Ding et al.^11^ and we inject pertussis toxin intravenously instead of intraperitoneally. Altogether, based on the present studies, depending upon one’s needs, ventral immunization could be used for a rapid induction of NMDARE, whereas dorsal immunization and repeated boosting could be utilized for more chronic, longitudinal studies.

In this work, we present the 4 week ventral immunization protocol as a translational model of NMDARE. Patients with NMDARE typically develop symptoms including psychosis, confusion, seizures, movement disorders, hypoventilation, dysautonomia, and coma (1). Longitudinal neuropsychologic profiling of NMDARE patients (26, 27) has demonstrated that they have dysfunction of the temporolimbic and frontal lobe pathways, manifesting as memory deficits, dysexecutive function, and neuropsychiatric changes, including depression and anxiety. Our chosen behavioral tests reflected tests that had been used to test mice in the first adoptive transfer mouse model of NMDARE (6), as well as the comparison active immunization models of NMDARE used for comparison (12, 13). We utilized additional tests, especially in later stages, when differences could be harder to detect, the further away the mice were from immunization. We were able to demonstrate changes in measures of memory, depression, and anxiety in the immunized mice, mirroring memory deficiencies and neuropsychiatric symptoms seen in NMDARE patients. While we did not see overt movement disorders in the mice, some dorsally-immunized mice demonstrated increased repetitive grooming movements during testing, which impacted their performance on the open field, Y-maze, and locomotor testing. Spontaneous seizures were not observed in immunized mice. A decreased seizure threshold (28) and spontaneous seizures (29) were demonstrated in adoptive transfer models of NMDARE in rodents. Future studies into the ventrally-induced active immunization model should also incorporate an electrographic assessment. Hypoventilation, dysautonomia, and/or decreased consciousness have not previously been described in mouse models of NMDARE. We observed that some immunized mice were slower to wake up from anesthesia after retro-orbital bleeding when compared to non-immunized control mice, which could be a possible manifestation of these conditions. Future studies should be designed to quantify objective differences in these physiological states between immunized and control mice.

Beyond behavior, a strong translational model for NMDARE also needs to provide immunologic insights. Our work demonstrated changes in blood and intracerebral immune cells and cytokines in immunized mice. Patient-derived pathologic specimens in NMDARE are rare, as the majority of patients survive. An early paper on NMDARE (30) mentioned 12 out of 14 patients’ brain biopsies demonstrating nonspecific mild perivascular lymphocytic cuffing. A more recent neuropathologic analysis of four brains from NMDARE patients (4) included two untreated patients. Their hippocampi showed decreased NMDAR immunoreactivity, correlating with disease severity. Their brains also had perivascular and parenchymal infiltrates of CD3+/CD8- T cells and plasma cells in their basal ganglia, amygdalae, and hippocampi, potentially accounting for observed movement disorders, neuropsychiatric symptoms, and memory deficits. Ventrally-induced mice also demonstrated decreased hippocampal NMDARs and intracerebral T cells and plasma cells 4 weeks after immunization. A recent metanalysis of cytokines from the blood and spinal fluid of NMDARE patients (5) demonstrated a predominance of T-cell-associated cytokines, along with some B-cell-related and broad-spectrum cytokines in the CSF of NMDARE patients, as compared to controls. There were elevations of IFN-γ, IL-1β, TNF-α, IL-6, IL-10, and IL-12 noted in NMDARE patients’ spinal fluid, cytokines which were included in the commercial pro-inflammatory kit we used. Our studies measured cytokines in brain tissue and not CSF, making it difficult to make direct comparisons. However, elevations in TNF-α were seen in ventrally-induced mice at 2 weeks, and elevations in TNF-α, IL-1β, and IL-6 were seen in the brains of dorsally-induced mice at 6, 8, and 14 weeks post immunization, respectively. While it is challenging to correlate behavioral with immunologic changes, analyzing our 4 week flow cytometry data, when ventrally-induced mice were demonstrating memory deficits and depressive behavior, in contrast to dorsally-induced mice, they had intracerebral T cells in addition to plasma cells, consistent with patient-based literature, which in particular seems to implicate CD8- (CD4+) T cells in NMDARE.

There are limitations to the present work. First, EAE uses the powerful immune adjuvant *Mtb* and co-adjuvant pertussis toxin to overcome tolerance and elicit an autoimmune response. It is well known to trigger a prominent T-cell-mediated immune response (31), possibly related to the use of *Mtb*. Thus, although NMDAR Abs are produced in the blood and spinal fluid (**Figure 2**) and plasma cells migrate into the brains of immunized mice (**Figure 5**), the role of T cells in this mouse model may be exaggerated, as compared to that in NMDARE patients. Future work could try to use incomplete Freund’s adjuvant or a different oil adjuvant (32) that lacks mycobacteria or inject the NMDAR ATD peptide directly into the peritoneum or thymus of mice, without immune adjuvants (33). Second, plasma cells were difficult to find in the brains of immunized mice, as assessed via flow cytometry. This may be because meninges and dura, where plasma cells have been found in patients with multiple sclerosis (34), were excluded from our tissue preparation. Future work could focus on additionally extracting dura, meninges, skull bone marrow (35), and extracranial lymph nodes (36) to enrich for plasma cells. Third, including the control group, we had 4 different groups for comparison. Given the large number of treatment groups, control mice were naïve, as opposed to sham-induced to match each of the 3 methodologies being tested. Also, given the large number of mice in the 8 and 14 week time points, novel object testing parameters were trimmed. This may have contributed to there being a lack of significant difference between treatment groups and naïve mice, as the discrimination index for the control mice at 14 weeks, in particular, was less than expected. Lastly, as the immunized mouse brains were utilized for many investigations, some of the experiments, such as cluster density or flow cytometry analysis, had fewer samples. Multiple smaller areas were sampled within the imaged hippocampal regions to increase the representation of each brain for cluster analysis and additional control mouse brain hemispheres were added to the flow cytometry experiments to enhance differences from naïve controls. Future studies could pare down the overall number of groups, include sham-induced mice, and include larger numbers of mice within each group.

The present work establishes the conditions for a relatively rapid induction of an NMDARE phenotype in mice from behavioral, biochemical, and immunologic perspectives. This, in turn, allows for more mechanistic studies into the pathophysiology of autoimmune encephalitis, identifying avenues for the development of novel diagnostic biomarkers and therapeutic targets for this devastating disorder. The 4 week ventrally-immunized NMDARE mouse model could also serve as an efficient translational platform for pre-clinical studies for the testing of existing and future therapeutic interventions.

## Data availability statement

The raw data supporting the conclusions of this article will be made available by the authors, without undue reservation.

## Ethics statement

This animal study was reviewed and approved by the Institutional Animal Care and Use Committee, Massachusetts General Hospital, Boston, MA, USA.

## Author contributions

JL contributed to study concept and design, implementation of experiments, acquisition of data, analysis and interpretation of data, and writing of manuscript. NJM contributed to study concept and design, implementation of experiments, acquisition of data, analysis and interpretation of data, and critical revision of manuscript. GJ contributed to implementation of experiments, acquisition of data, analysis and interpretation of data, and critical review of manuscript. C-CL contributed to study concept and design, implementation of experiments, acquisition of data, analysis and interpretation of data, and critical review of manuscript. EK contributed to study concept and design, implementation of experiments, and critical review of manuscript. GW contributed to implementation of experiments, and critical review of manuscript. RT contributed to study supervision and critical review of manuscript. JC contributed to study concept and design, study supervision, analysis and interpretation of data, and critical review of manuscript. All authors contributed to the article and approved the submitted version.
