# Supplementary material for: Optimizing animal models of autoimmune encephalitis using active immunization
Source: Front Immunol. 2023 Jul 12;14:1177672. doi: 10.3389/fimmu.2023.1177672 (PMC10374403; doi:10.3389/fimmu.2023.1177672)
Supplement: Supplementary file 1 [file DataSheet_1.pdf]

Supplemental Material

Methods:

Sample Size Calculation: prior to performing the experiments outlined in this manuscript, we carried out a pilot study with the same treatment groups to 12 weeks post-immunization. Based on flow cytometry results from our pilot study, examining the number of CD4+ T-cells in the brain at 12 weeks post-immunization, ventrally-induced mice had a mean of 4.073 cells/mg brain tissue with a standard deviation of 0.238 (which was roughly equal to the mean for a smaller number of control mice), while dorsally-immunized boosted mice had a mean of 9.214 cells/mg brain tissue and a standard deviation of 1.44. With the parameters of 80% power and an alpha level of 0.05, *the estimated minimum number of mice needed for a 12-week study was calculated as 6 mice per group*. When using 10 mice per group and an alpha of 0.05, the power increased to greater than 99%. In the experiments outlined in this manuscript, we utilized 10 mice per group for the 8-week and 14-week mice, which is also supported by data published by Ding et al.[1] LTP experiments carried out in hippocampal slices from immunized mice by Ding et al. showed that with group means of 143.1% and 112.0% normalized field EPSP and respective standard deviations of 12.3 and 13.2 for control and boosted mice, using the parameters of 80% power and an alpha of 0.05, *the estimated minimum number of mice needed was calculated as 10 mice*. This supports our use of 10 mice per group for our 8 and 14 week inductions. Similar to the combined serum titer data from all weeks (**Figure 2B**), our pilot data also showed that the peak NMDAR Ab titer was at 6 weeks post-immunization. As detailed above, sample size calculations based on our pilot study showed that 6 mice should be sufficient to capture the effects of immunization at 12 weeks post-immunization with 80% power and an alpha of 0.05. At 12 weeks, it is expected that the effects of immunization in unboosted groups are wearing off, as

was seen with the ventrally-induced mice in the pilot project. Thus, we expected that at the presumed peak of the disease time course (as judged by maximal NMDAR Ab levels at 6 weeks post-immunization), the effects of immunization would be larger and more apparent than at 12 weeks and 5 mice should be sufficient to capture these effects. Therefore, for the 2, 4, and 6-week inductions, 5 mice per group were used.

Immunization with GluN1 ATD peptide

Prior to immunization, mice were acclimated to their holding room for over a week after arrival from a commercial vendor. They were a standard strain and were naïve to procedures prior to their pre-immunization bleeds, which were performed in our animal facility. Mice were housed in cages of 5 with other mice in the same induction group. 8-week-old female C57BL/6 mice (Jackson Laboratories, Bar Harbor, ME; 5 per group for 2, 4, and 6-week timepoints and 10 per group for 8 and 14-week timepoints) were anesthetized with 3% isoflurane. Boosted dorsally-induced mice were immunized as per Ding et al[1]. Briefly, a one-to-one emulsion was prepared of 1) NMDAR GluN1 ATD peptide (LQNRKLVQVGIYNGTHVIPNDRKIIWPGGE) dissolved in sterile PBS and 2) Complete Freund's Adjuvant (CFA, Sigma, St. Louis, MO) supplemented with *Mycobacterium tuberculosis* H37Ra (*Mtb*, 8 mg/mL, BD Difco, Franklin Lakes, NJ) at a final peptide concentration of 2 mg/mL. The emulsion components were prepared in separate 1 mL glass syringes, which were connected via a 20-G metal needle connector, and an emulsion was prepared by transferring between the two syringes[2]. 200 μg peptide was injected per mouse via two 50 μL subcutaneous injections of emulsion into either side of the tail base. Mice received two booster injections of peptide emulsion mixture 4 and 8 weeks after initial immunization. At the last immunization as well as 48 hours later, mice were injected intraperitoneally with 200 ng of pertussis toxin (Sigma) dissolved in sterile PBS.

Immunofluorescent cell based assay (CBA) for NMDAR antibody detection

The presence of NMDAR antibodies in mouse serum and CSF was determined using a commercial NMDAR transfected HEK293 CBA kit (FB 112d-1010-51, Euroimmun, Lübeck, Germany), with slight modifications to the manufacturer's protocol, as detailed. In brief, mouse serum or CSF was diluted with 0.2% Tween-20 in PBS (PBST) containing a commercial rabbit antibody to GluN1 (1:1250, G8913, Sigma) and was applied to wells for 30 minutes at room temperature (RT). Slides were briefly rinsed and then washed for 5 minutes with shaking in PBST. Dylight© 488 (1:200 in PBS) and 649 (1:750 in PBS) secondary antibodies against mouse and rabbit IgG (BioLegend, San Diego, CA) were applied to the wells for 30 minutes at RT. Slides were briefly rinsed and washed for 5 minutes in PBST with shaking. Mounting media and a coverslip from the CBA kit were then applied to the slides, and fluorescent signal was captured with an Andor camera (Oxford Instruments) mounted on an epifluorescence microscope (Eclipse Ci, Nikon, Melville, NY) using Nikon NIS-Elements software. Titers were ascertained by sequentially diluting samples and determining the last concentration at which NMDAR staining was observed (as confirmed by colocalization with the commercial GluN1 antibody).

Animal behavioral tests

Mice were housed in windowless animal facilities with a 12-hour light/dark cycle. To minimize potential confounders, mice were acclimated to their holding room for over a week before any handling was performed. To minimize stress to the mice, they were primarily handled by JL and GJ throughout the experiment. Testing was done by GJ and JL according to standardized protocols. Care was taken to avoid stressful procedures, such as anesthesia and retroorbital bleeding, during behavioral testing periods. Mice were housed in the same room during the entire experiment and before behavioral experiments, mice cages were brought to a

specialized behavioral room and mice were allowed to acclimate in their home cages for at least 30 minutes before experiments were started. The behavioral equipment was cleaned thoroughly between each mouse and treatment group, to ensure that mice were not influenced by the smell of other mice. To minimize sampling confounding effects, testing and procedures were generally carried out at the same time of day and behavioral testing was generally done in the same treatment group order across the entirety of the experiment.

Novel object recognition (NOR; memory):

NOR testing was done in an "open field" paradigm as previously described[3]. Briefly, mice were habituated by allowing them to freely roam for 30 minutes in an empty clear plexiglass box (30 cm wide x 22.5 cm depth x 31 cm height) custom built for mouse behavioral testing with a video camera mounted on the top. The next day, mice were placed into the box for 9 minutes during familiarization with two identical objects at the "northeast" and "southwest" corners of an imaginary box (14 cm x 10.5 cm) centered at the center of the box. After a retention period of 3 hours spent in their home cage, the mice were placed back into the box for 9 minutes for the testing trial, with objects placed at the opposite ("northwest" and "southeast") corners. One of the familiar objects (half of the times on the left and the other half on the right) was replaced with a novel object, and the time spent exploring each object (familiar and novel) was recorded. A discrimination index was calculated as time spent exploring the novel object minus time spent exploring the familiar object divided by total time spent exploring both objects. Due to time constraints with the greater number of mice at the 8 and 14-week timepoints, the habituation time was cut to 10 minutes, the retention time was cut to 2.5 hours, and the familiarization and testing phases were each cut to 5 minutes.

Barnes maze (memory)[4]:

The Barnes maze consists of a raised circular surface with 20 circular holes around its circumference. 4 visual cues (colored shapes) were evenly spaced and suspended around the maze in plain sight of the animal. The table surface is brightly lit by overhead lighting. Under one of the holes is an "escape box". During habituation, mice were placed under a small box in the center of the maze for 10 seconds. The box was then lifted, and they were guided to the correct hole and introduced into the escape box, which was covered for 2 minutes. At the 8-week timepoint, all mice in a cage were sequentially habituated before starting their training trials on the same day. At the 14-week timepoint, mice from all cages were sequentially habituated before all mice began their training trials on the same day. During training trials, mice were placed into the box at the center of the maze. After 10 seconds, they were allowed up to 3 minutes to find and enter the correct hole. They were encouraged to enter the hole with a gentle tail pull, if they did not spontaneously enter it. If they did not find the hole within 3 minutes, they were led to it and encouraged to enter it. Once in the hole, it was covered for 1 minute. Due to time constraints given the large number of mice per group, mice underwent a shortened Barnes maze protocol[5] consisting of 2 days of training (the first one included habituation first), during which each cage had 3 consecutive training rounds. Probe trials were conducted a day after completing the training. During the probe trial, all holes, including the escape hole, were closed. Mice were placed into the box at the center of the maze. After 10 seconds, they were given 90 seconds to explore the maze. The time it took for mice to find the former site of the escape hole (now closed) was recorded.

Y maze (memory)[6]:

Mice were placed into the center of a plastic Y-shaped maze with three arms (30 cm length x 9 cm width x 13 cm height) at a 120 angle from each other, with one of the arms closed off. They were allowed to freely roam for 5 minutes. After a retention period of 30 minutes[7] or 2.5 hours[8], the mice were placed back into the center of the apparatus, with all arms open, where they were allowed to freely roam for 5 minutes. A retention period of 2.5 hours was used for all mice at the 8-week timepoint. A retention period of 30 minutes was used for half of the mice and a retention period of 2.5 hours was used for the other half of the mice at the 14-week timepoint (**Supplemental Figure 1B**), given that naïve control mice did not perform the task as expected at the 8-week timepoint. The number of entries and time spent per arm (both raw and as a percentage of total), total arm entries, and total exploration time were tabulated.

Fear conditioning (learning/memory)[9]:

Mice were trained and tested on 2 consecutive days. On the training day, mice were placed into a lit 29 cm wide x 24 cm depth x 24 cm height chamber (Med Associates Inc., Albans, VT) with solid walls and steel metal grate flooring for a total of 6.5 minutes. For the first 3 minutes, they were allowed to freely roam and baseline freezing activity was tabulated. Then an auditory cue was played for 15 seconds. During the last second of the tone presentation, a 0.7 mA shock was applied to the metal floor. Mice were then allowed to roam for 1 minute, after which the auditory cue was repeated for 15 seconds, and the electric shock was repeated during the last second of the tone. Mice were again allowed to roam for 1 minute, after which the auditory cue was repeated for 15 seconds, and the electric shock was repeated during the last second of the tone. Mice were allowed to recover for 45 seconds in the testing chamber before being removed. Contextual memory was tested 24 hours after training. The mice were placed

into the same chamber for 4 minutes with no tones or shocks. Freezing activity (number of freezes and percentage of time spent frozen) and number of bowel movements were tabulated. Cue testing (7 minutes total) was conducted after 3 hours. The mice were placed into the same chamber with different color and texture papers placed onto the walls and floor (covering the grate floor). For the first 3 minutes, they were allowed to freely roam. For the next 3 minutes, the same auditory tone as the day before was applied. For the next minute, they were allowed to recover before being removed from the testing chamber. Freezing activity (number of freezes and percentage of time spent frozen) and number of bowel movements were tabulated.

<u>Open field (anxiety)[10]:</u>

During the first 10 minutes of the habituation phase for NOR testing (above), the time to first enter, number of times, and the amount of time spent by mice in a central imaginary box (14 cm x 10.5 cm) were tabulated.

<u>O-maze (Zero-maze; anxiety)[11]:</u>

Mice were placed on a raised black opaque plexiglass circular platform with an open center (inner diameter 50 cm, outer diameter 55.5 cm), two opposite open quadrants, and two opposite enclosed quadrants (walled, 19 cm height). The platform was 60 cm above floor level, with a 5.5 cm wide circular path through the quadrants. Mice were placed into an enclosed quadrant at the start of the test and allowed to explore the maze for 5 minutes, recording the number of times and time spent in the open quadrants.

<u>Forced swim test (depressive behavior)[12]:</u>

Mice were placed individually into an 18 cm diameter glass cylinder filled with 15 cm water kept between 23 - 25°C for 6 minutes. Total immobile time was recorded beginning after 1 minute, defined as the mouse floating with no active movement of any limbs or tail. After the

test, the mice were placed into a cage with absorbent paper towel, and then returned to their home cage once they were dry.

Tail suspension (depressive behavior)[13]:

A cut thin plastic straw (1 cm diameter and 4 cm length)[14] was placed over the mouse's tail to prevent tail climbing behavior. Mice were suspended from a horizontal pole 50 cm above a table using adhesive tape placed 3/4 from the tail base. For 6 minutes, the total time of immobility was recorded.

Nesting (general wellbeing)[15]:

Approximately 1 hour prior to the evening dark phase, animals were singly housed in cages with normal bedding and a 3-gram standardized square nestlet (Ancare, Bellmore, NY) of nesting material. 15 hours later, after an overnight phase, their nests were rated on a scale of 1 to 5 and the unused nestlet material was weighed, in order to calculate the untorn percentage. Mice were returned back to cages with their original litter mates at the conclusion of the testing. 2 and 4-week timepoint mice were not habituated ahead of testing. Habituation was noted to increase the nesting scores and decrease the percentage of untorn nestlet for control mice. Spanning over a few days before testing, socially housed mice at the 6, 8, and 14-week timepoints were given a square nestlet instead of their regular nesting material to become acquainted with it.

Locomotor activity (motor activity):

The custom built clear plexiglass behavioral box used for NOR testing was equipped with an external grid of infrared photocells placed 1 inch apart and 2 cm above the floor to evaluate horizontal movements. The movement of the mice during the first 10 minutes of the NOR habituation period was recorded, and custom software was used to calculate the animals' speed (distance/time).

Immunofluorescence and confocal microscopy for NMDAR cluster density analysis

Frozen lightly fixed and sucrose-protected half brains (2 per group for 2, 4, and 6-week timepoints, 3 per group for the 8-week timepoint, and 5 per group for the 14-week timepoint) were sectioned into 10 μm sagittal slices and mounted onto TruBond™ 380 slides (Electron Microscopy Sciences, Hatfield, PA). Slices were rehydrated with PBS for 10 minutes before blocking with 5% goat serum and 1% BSA in PBS for 60 minutes at RT. They were then incubated with human CSF containing NMDAR antibodies (ARUP Laboratories, 1:20 in blocking buffer) overnight at 4°C. Slides were washed for 3 x 5 minutes with cold PBS, incubated with anti-human IgG Alexa Fluor™ 647 (1:50 in PBS, clone A21445, Thermo Fisher) for 60 minutes at RT, washed 3 x 5 minutes with cold PBS, permeabilized with 0.3% Triton™ X-100 for 10 minutes at RT, washed 3 x 5 minutes with cold PBS, blocked for 60 minutes at RT, and incubated with rabbit anti-PSD95 antibodies (1:150 in blocking buffer, clone 18258, Abcam) overnight at 4°C. Slides were washed 3 x 5 minutes with cold PBS and incubated with Cy™3-conjugated AffiniPure goat anti-rabbit IgG (1:200 in PBS, 111-165-144, Jackson ImmunoResearch Laboratories, West Grove, PA) for 60 minutes at RT. They were then washed 3 x 5 minutes with cold PBS, and mounted with ProLong™ Diamond Antifade mountant (Molecular Probes, Eugene, OR) containing DAPI (1:5000, Thermo Scientific).

Slides were scanned with a Leica SP5 confocal microscope, 63X oil objective with 3X zoom. 3 μm z-stacks sampled every 0.1 μm were acquired from two hippocampal dentate gyrus and two CA1 regions at 1024 x 1024 lateral resolution (82 μm^2). Images were deconvolved using standard confocal settings in Huygens Essential software (Scientific Volume Imaging, Hilversum, Netherlands). Imaris Suite 9.8.2 (Oxford Instruments) was used to sample three smaller 256 x 256 lateral regions (20.5 μm^2) within each deconvolved z-stack. A spot detection

algorithm was used to quantify NMDAR and PSD-95 clusters in the sub-stacks. A three-dimensional spot co-localization algorithm (within 0.2 µm) was used to quantify postsynaptic NMDAR clusters.

Flow cytometry

Half brains were removed from ice-cold PBS and were mechanically disrupted with a glass Dounce homogenizer and passed through a 40 µm nylon cell strainer (BD Biosciences, San Jose, CA, USA). Infiltrating immune cells were isolated from the interface of a 30/70 Percoll (GE Healthcare, Boston, MA, USA) gradient after centrifugation for 5 minutes at 350 g at 18°C and resuspended in 150 µl FACS buffer (PBS with 0.5% BSA (MP Biomedicals, Solon, OH)) for surface antibody staining. Antibody panels are as described below. All antibodies were from BioLegend and were used at a concentration of 1:200, unless otherwise noted. Briefly, cells were first incubated on ice with anti-CD16/CD32 (1:100) to block Fc binding sites for 20 minutes and were then washed, spun (5 minutes at 350 g at 4°C), and resuspended with 150 µl FACS buffer 3 times and were stained with anti-mouse antibodies against CD11b (APC7), CD45 (AF700), CD3 (AF647, 1:100), CD4 (AF488), CD8a (PE7), B220 (BV421), TACI (PE, 1:100), CD138 (PerCp5.5, 1:100), and LIVE/DEAD Zombie Aqua™ Fixable Viability Kit (1:200). Staining was done for 30 minutes on ice, followed by a wash, spin (5 minutes at 350 g at 4°C) and resuspension in 150 µl FACS buffer. Cells were then fixed with fixation buffer (Biolegend) for 20 minutes on ice. Blood was lysed in red blood cell lysis buffer (Biolegend), spun (5 minutes at 350 g at 4°C), resuspended in 150 µl FACS buffer, and stained as above. Spleens were harvested into homogenization buffer (PBS + 5% BSA + 2 mm EDTA (Sigma)) and digested in an enzymatic mixture (2.5 mg/mL Collagenase D (Millipore Sigma)) for 30 minutes at RT, with gentle shaking every 10 minutes. The mixture was then filtered through a cell strainer, spun

down (5 minutes at 350 g at 4°C) for resuspension in 150 µl FACS buffer, and stained as above. For leukocyte analysis, cells were first analyzed via single cell and live / dead identification. Leukocytes were next identified via gating on CD45+. Cells were further divided into B cell and T cell populations by gating for B220+ (B cell specific) and CD3+ (total T cell specific). CD3+ cells were subdivided into CD4+ (T helper) and CD8+ (T cytotoxic). Plasma cells were identified by TACI and CD138 positivity. All events were acquired on an LSRII flow cytometer (BD Biosciences) and analyzed with BD FlowJo software (version 10.4). Results were tabulated as percentage of live cells.

Cytokine analyses

Briefly, 50 µL of thawed undiluted sample and calibrator diluted in RIPA buffer containing protease inhibitors were added in duplicate to wells coated with capture antibodies to IFN-γ, IL-1β, IL-2, IL-4, IL-5, IL-6, IL-10, IL-12p70, KC/GRO (CXCL1), and TNF-α and were incubated for 2 hours at RT on a 96-well plate shaker (780 rpm). The plate was washed x 3 and 25 µL of antibody detection solution was added to the wells before incubation for 2 hours at RT on a plate shaker (780 rpm). The plates were washed x 3 before adding 150 µL 2X MSD Read Buffer, immediately read on a Meso QuickPlex SQ 120 system (MSD), and analyzed using MSD Discovery Workbench (MSD) software. Results (as pg / mL) were checked against the threshold of detection for the plate, before being converted to pg / g brain tissue.

Survival

Four of 130 total allotted mice died during the course of the experiments. One ventrally-induced mouse from the 4-week group died during intravenous injection of pertussis toxin after subcutaneous injections of CFA/peptide emulsion. It was replaced by a sex and age-matched mouse from a different litter. One dorsally-induced boosted mouse from the 8-week group died

at 4 weeks, during boosting. One dorsally-induced unboosted mouse from the 14-week group died during its pre-immunization retro-orbital bleed. Another dorsally-induced unboosted mouse from the 14-week group was euthanized at 7 weeks post-immunization due to orbital damage from repeated retro-orbital bleeds. Thus, 126 + 1 replacement mouse completed the experiments.

Supplementary Table 1:

Serum:

	**Ventral**	**Dorsal**	**Dorsal-Boosted**
**2 weeks**	35	35	25
**4 weeks**	30	29	25
**6 weeks**	15	14	15
**8 weeks**	20	18	19
**14 weeks**	10	8	10

CSF (non-bloody samples):

	**Ventral**	**Dorsal**	**Dorsal-Boosted**
**2 weeks**	5	4	0
**4 weeks**	5	5	0
**6 weeks**	5	2	5
**8 weeks**	8	9	6
**14 weeks**	9	6	9

Supplementary Table 1 legend: **NMDAR Ab titering mouse numbers**. The number of mice per group per timepoint are detailed for the serum samples shown in **Figure 2B** (at least 8 per group) and the CSF samples shown in **Figure 2C** (at least 2 per group).

Supplementary Figure 1:

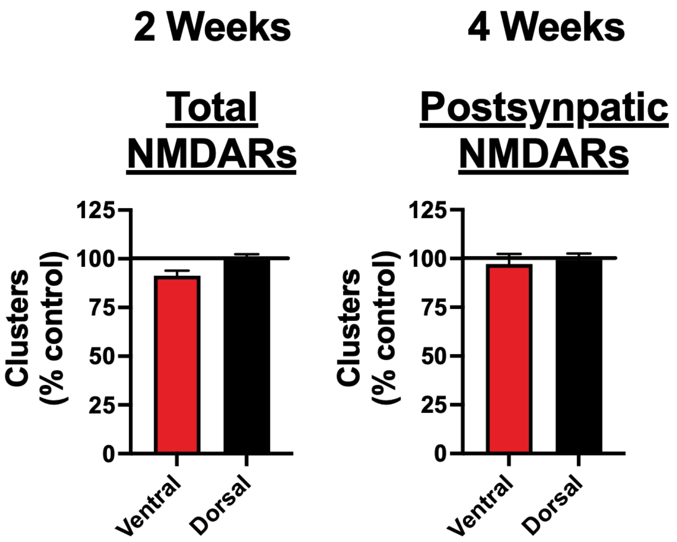

Supplementary Figure 1 legend: **Hippocampal total cell surface and postsynaptic NMDAR cluster density.** Mean total cell surface NMDAR (2-week timepoint) and postsynaptic NMDAR (4-week timepoint) cluster density for each induction group (Ventral or Dorsal) as a percentage normalized to the mean of the unimmunized control group, which is represented as a horizontal line at 100%. Error bars show standard errors of the normalized means. Results were tested for normality. For normally-distributed data, a 1-way ANOVA with Tukey's multiple comparison test was performed to determine differences between group means. For non-normally-distributed data, a Kruskal-Wallis 1-way ANOVA with Dunn's multiple comparison test was performed to determine differences between group means. No treatment group mean was statistically different from the mean of the control group.

Supplementary Figure 2:

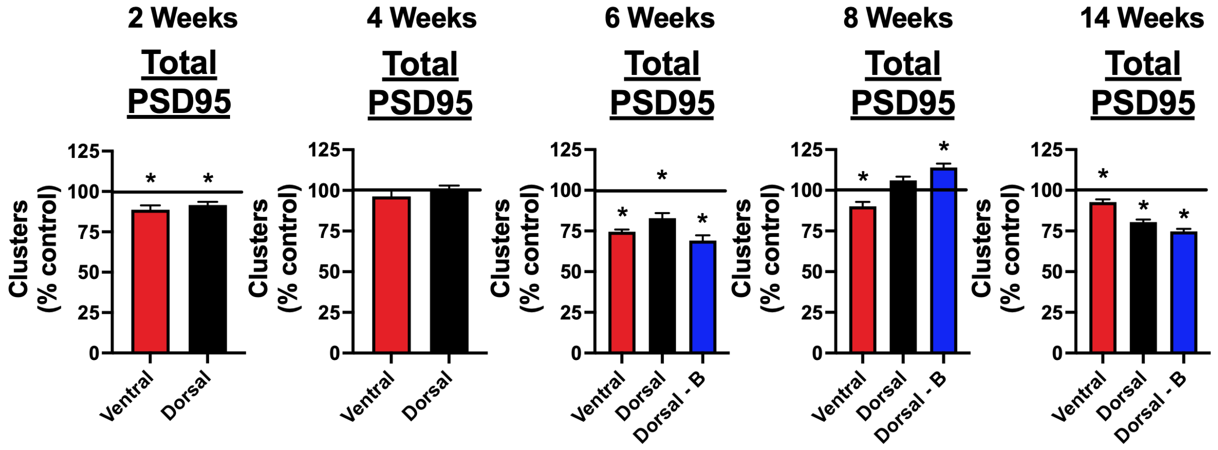

Supplementary Figure 2 legend: **Hippocampal total PSD95 cluster density.** Brains were fixed, cryoprotected, and frozen at 2, 4, 6, 8, and 14 weeks post-immunization. 10 µm sections were stained with human CSF containing NMDAR Abs, permeabilized, and then stained with a commercial Ab against PSD95. Mean total PSD95 cluster density for each induction group (Ventral, Dorsal, or Dorsal-boosted (Dorsal-B)) as a percentage normalized to the mean of the unimmunized control group, which is represented as a horizontal line at 100%. Error bars show standard errors of the normalized means. There were 2 stained half brains per group for the 2, 4, and 6-week timepoints, 3 stained half brains at 8 weeks, and 5 stained half brains per group at 14 weeks. Dorsally-boosted mice were only represented at 6 weeks and beyond, as boosting occurred at 4 weeks. Results were tested for normality. For normally-distributed data, a 1-way ANOVA with Tukey's multiple comparison test was performed to determine differences between group means. For non-normally-distributed data, a Kruskal-Wallis 1-way ANOVA with Dunn's multiple comparison test was performed to determine differences between group means. Graphs with at least one treatment group mean that was statistically different from the mean of the control group (signified by a '*') are displayed. *: $P < 0.05$.

Supplementary Figure 3:

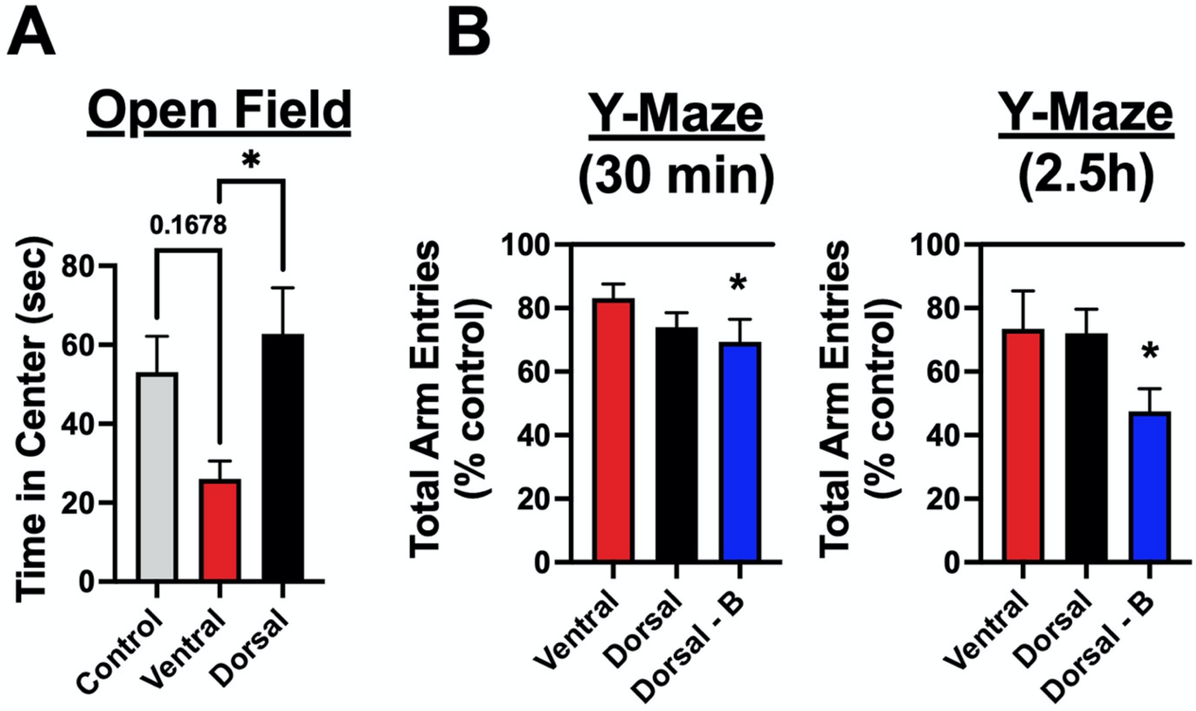

Supplementary Figure 3 legend: **A. Ventrally-immunized mice scored higher than dorsally-induced mice on a measure of anxiety at the 4-week timepoint.** One week prior to sacrifice, mice in the 4-week cohort underwent behavioral testing. They were tested for anxiety via open field assessment, as detailed above. There were 5 mice tested per group (Control, Ventral, and Dorsal), for the exception of 4 mice in the ventrally-induced group. One mouse in this group died during intravenous injection of pertussis toxin. Its replacement mouse was from a different litter and was housed with fewer litter mates. It was thus excluded from behavioral analyses. 1-way ANOVA with Tukey's multiple comparison test was performed to determine statistically significant differences between group means. Ventrally-immunized mice spent less time in the center of the open field than dorsally-induced mice at 4 weeks post-immunization, indicating a higher level of anxiety in these mice. **: $P <= 0.05$.* **B. Amotivational behavior in Y-maze task persisted in dorsal boosted mice whether pre-testing retention time was 30 minutes or**

2.5 hours at the 14-week timepoint. Mice underwent Y-maze behavioral testing as described above. One half of the 14-week timepoint mice were tested with a retention period of 30 minutes **(left)** and the other half had a retention period of 2.5 hours **(right)**. There were 5 mice tested per group (Control, Ventral, Dorsal, and Dorsal-boosted (Dorsal-B)), with the exception of 4 dorsally-induced unboosted mice, due to mouse deaths in this group. Data is shown as a mean percentage normalized to the mean of the unimmunized control group, which is represented as a horizontal line at 100%. Error bars depict standard error of the normalized mean. 1-way ANOVA with Tukey's multiple comparison test was performed to determine differences between group means. Dorsally-induced and boosted mice had fewer total arm entries than control mice regardless of the length of the retention period. *: $P < 0.05$.

Supplementary Figure 4:

A. 2-week timepoint

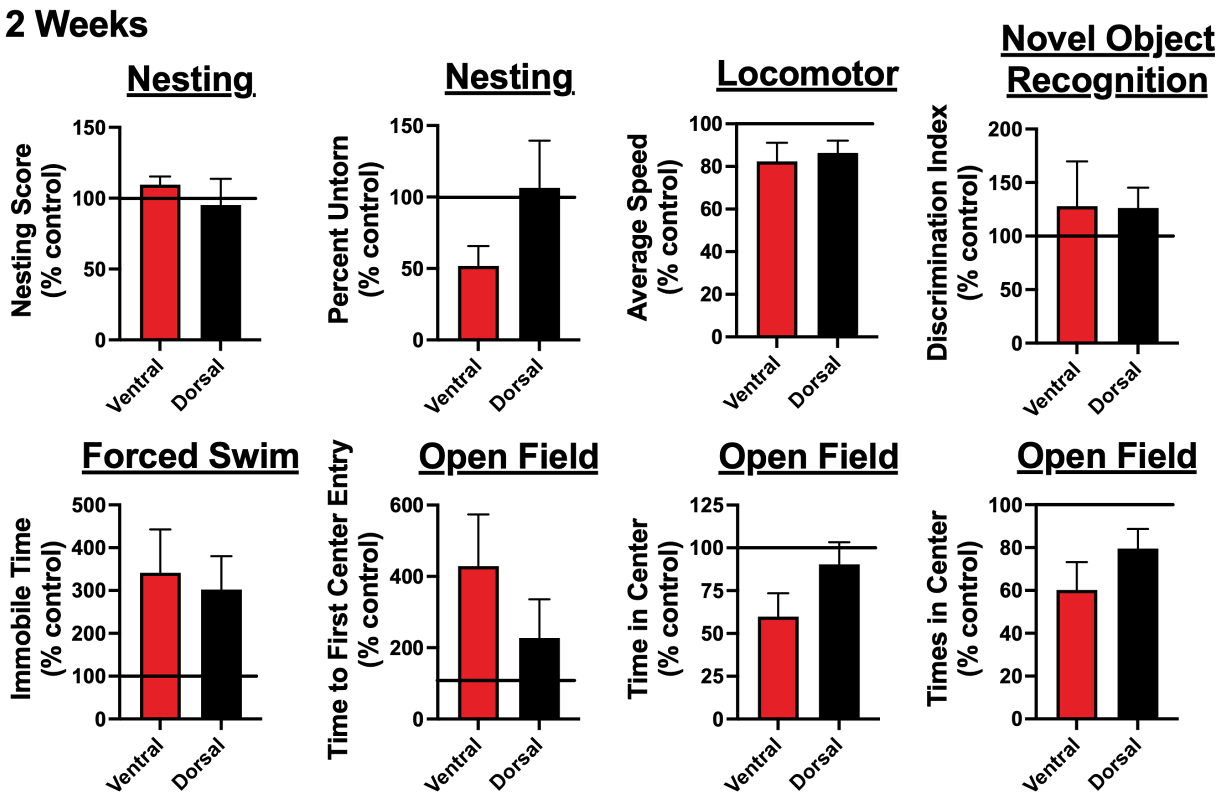

B. 4-week timepoint

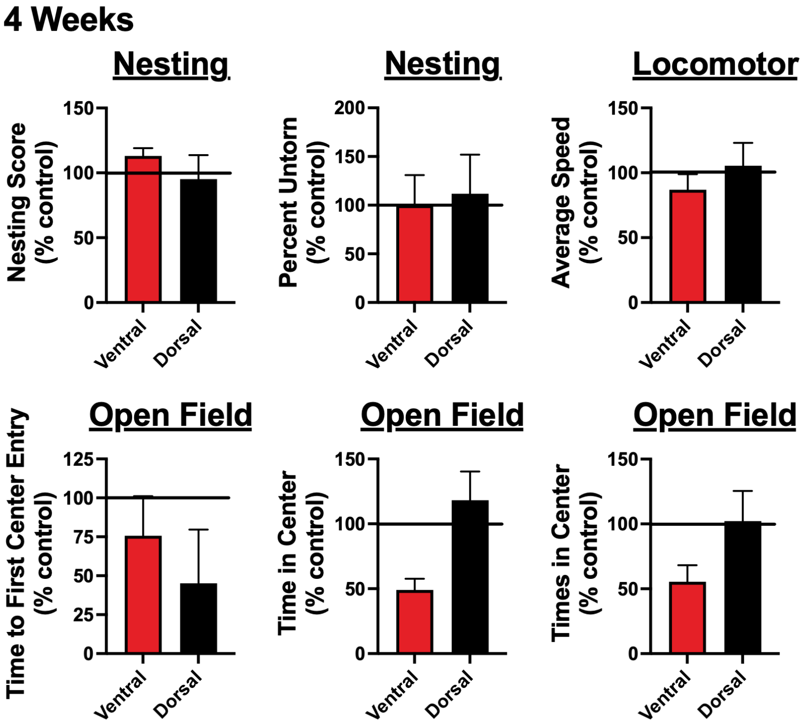

C. 6-week timepoint

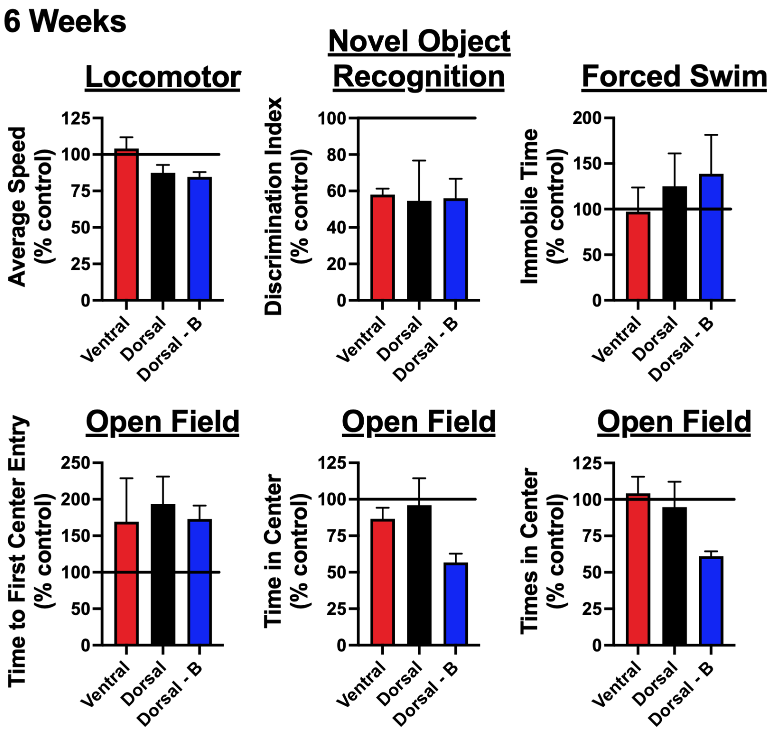

D. 8-week timepoint

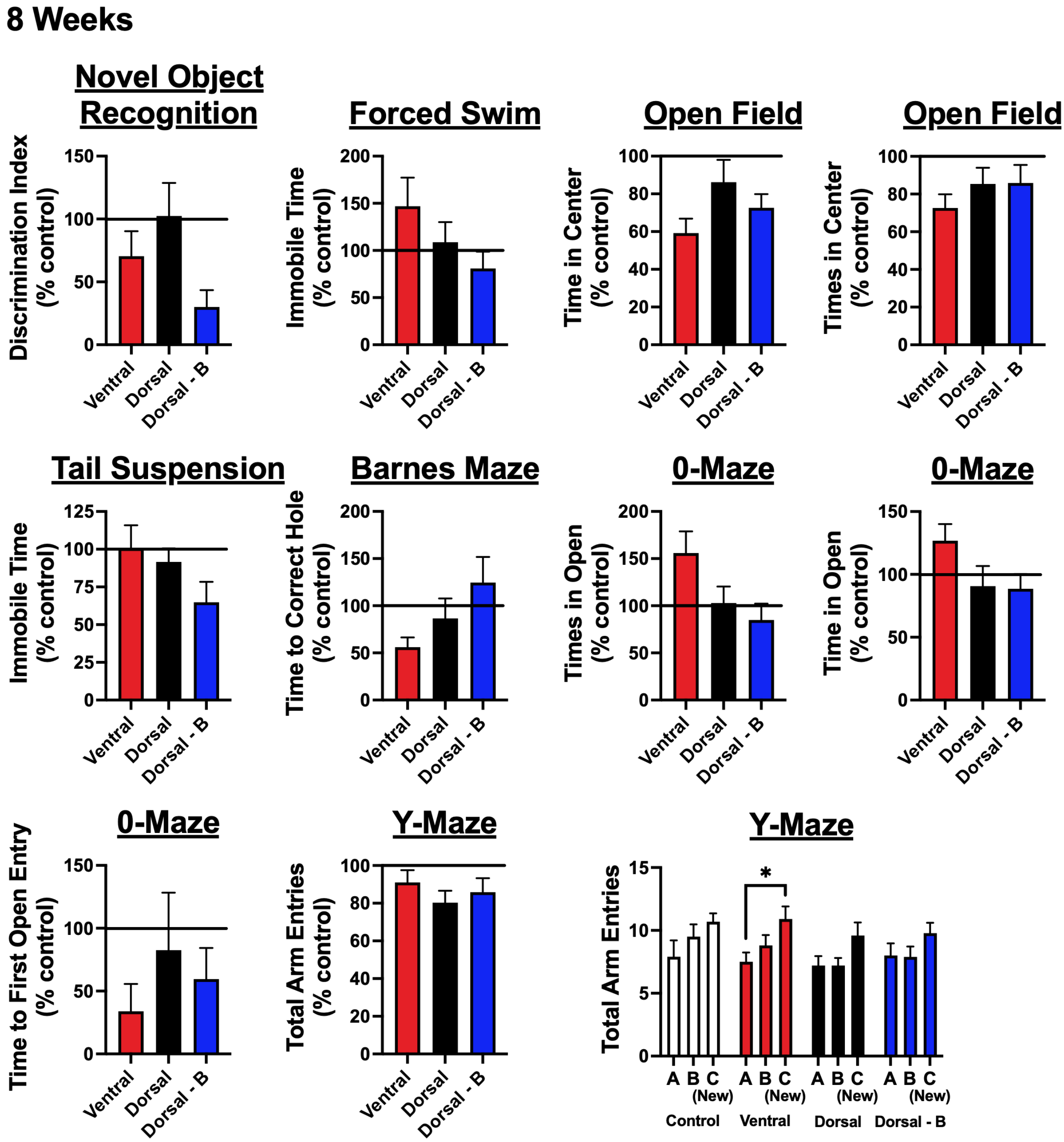

E. 14-week timepoint

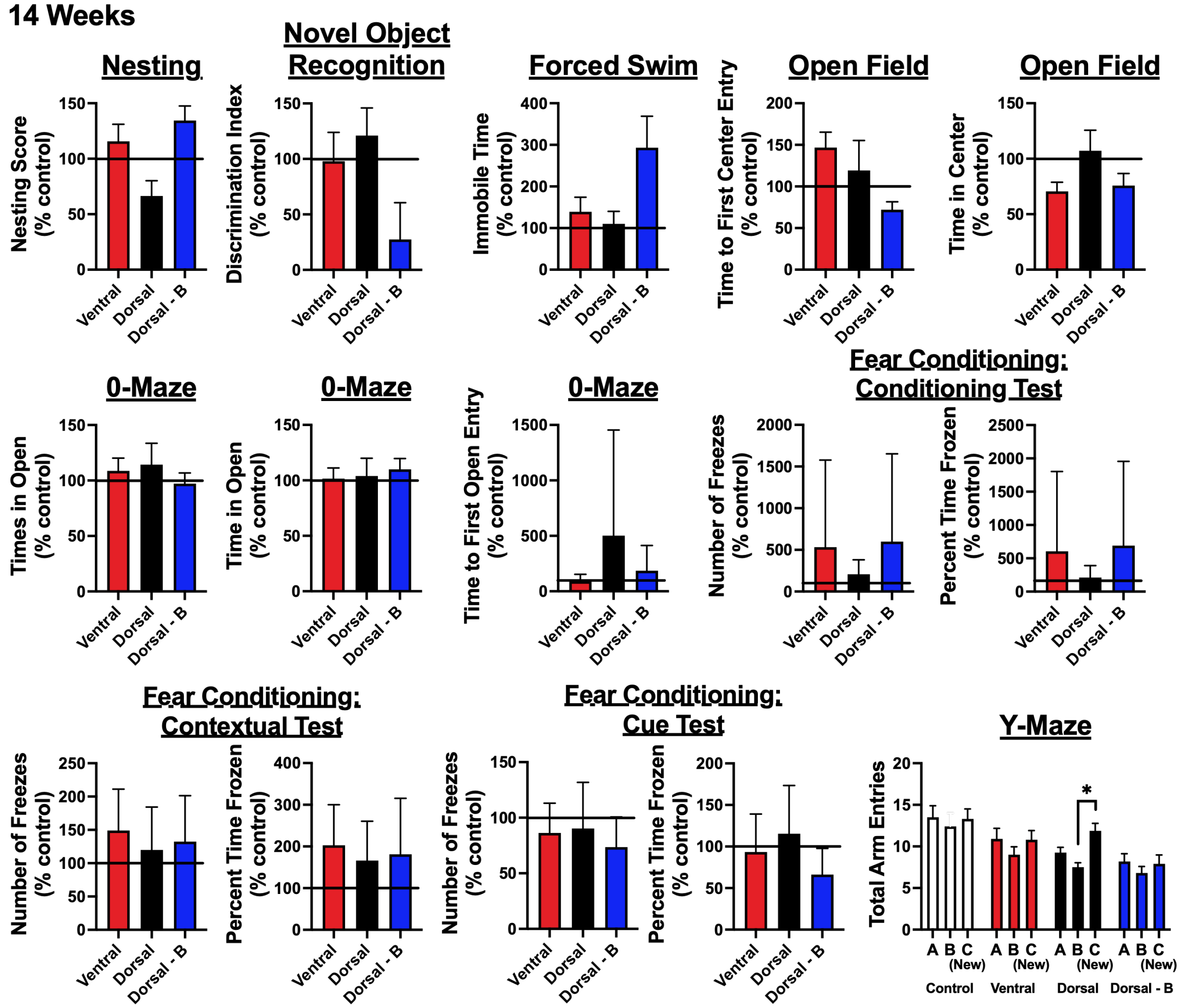

Supplementary Figure 4 legend: **Behavior testing results.** One (2, 4, and 6-week timepoints) to two (8 and 14-week timepoints) weeks prior to sacrifice, mice underwent behavioral testing. They were tested for memory deficits via novel object recognition (NOR) testing. Depression was assessed through forced swimming. Anxiety was tested for via open field assessment. General well-being was assessed through nest building. Motor and general function were assessed through locomotor monitoring. Additional memory tests, Y-maze and Barnes maze, and an additional test of anxiety, the O-maze, were carried out for the 8 and 14-week cohorts, which were tested after the 6-week peak of serum Ab titers (Figure 2B). An additional test for

depression, tail suspension, was carried out at the 8-week timepoint and an additional test for anxiety and memory, fear conditioning, was performed at the 14-week timepoint. There were 5 mice tested per group (Control, Ventral, Dorsal, and Dorsal-boosted (Dorsal-B)) at the 2, 4, and 6-week timepoints, for the exception of 4 mice at the 4-week timepoint in the ventrally-induced group, as one mouse in this group died during intravenous injection of pertussis toxin. Its replacement mouse was from a different litter and was housed with fewer litter mates. It was thus excluded from behavioral analyses. Boosted mice only had testing from 6 weeks and beyond, as boosting occurred at 4 weeks. There were 10 mice tested per group at the 8 and 14-week timepoints, with the exception of 9 dorsally-boosted mice at 8 weeks and 8 dorsally-induced unboosted mice at 14 weeks, due to mouse deaths in those groups. For the Barnes maze analysis, mice that demonstrated boredom with the task and did not attempt to look for the escape hole during the probe trial were excluded. This included 2 control mice at the 14-week timepoint. Results were tested for normality. For normally-distributed data, a 1-way ANOVA with Tukey's multiple comparison test was performed to determine differences between group means. For non-normally-distributed data, a Kruskal-Wallis 1-way ANOVA with Dunn's multiple comparison test was performed to determine differences between group means. No treatment group means were statistically different from the mean of the control group, although within the Y-maze results, there were some arm entry values which were statistically different from others within the same treatment groups, as denoted by '*'. *: $P < 0.05$. Aside from the Y-maze, data are shown as a mean percentage normalized to the mean of the unimmunized control group, which is represented as a horizontal line at 100%. Error bars depict standard errors of the normalized means.

Supplementary Figure 5:

A. 6 weeks blood

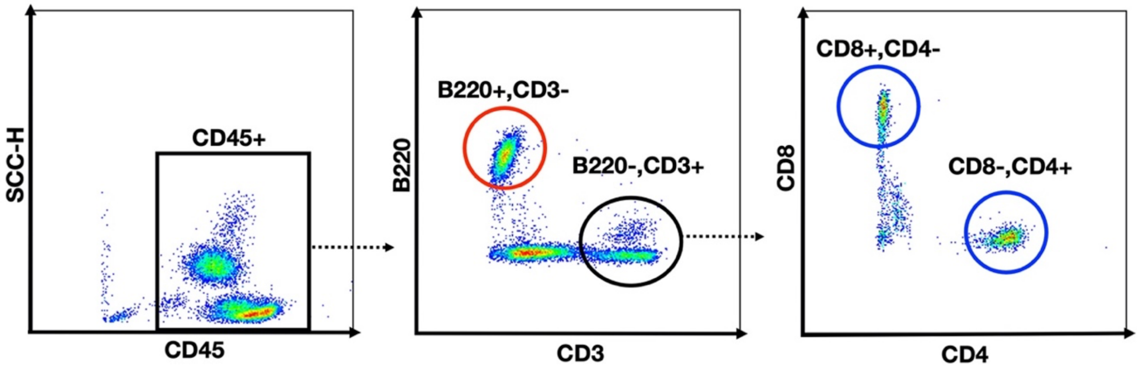

B. 4 weeks brain

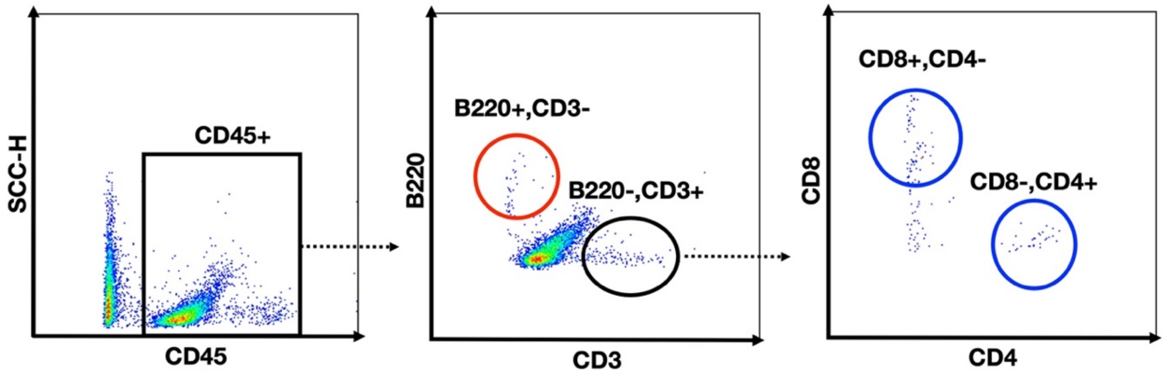

C. 4 weeks blood

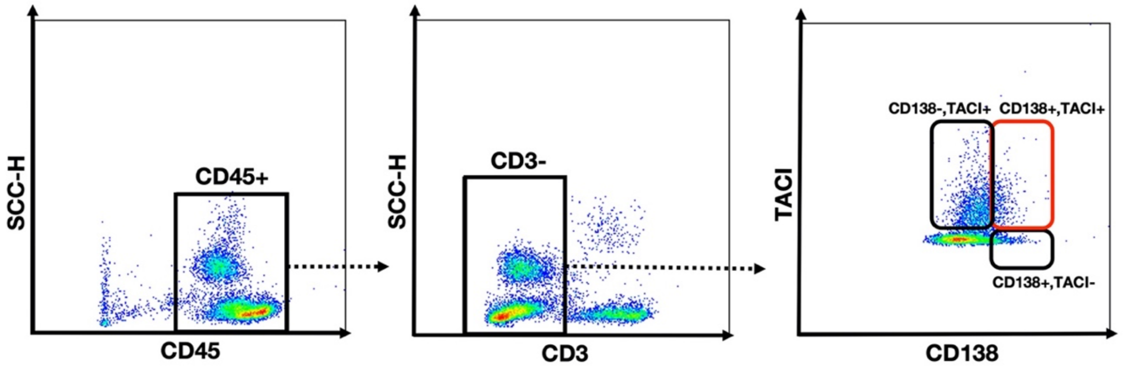

D. 4 weeks brain

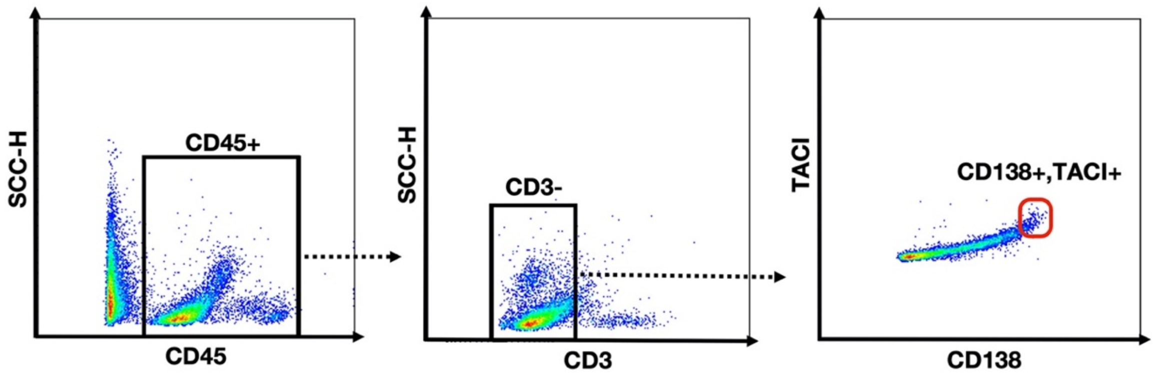

Supplementary Figure 5 legend: **Flow cytometry gating schemes for T cells, B cells, and plasma cells.** For leukocyte analysis, cells were first analyzed via single cell and live / dead identification. Leukocytes were next identified via gating on CD45+ (left panels). **A, B.** Cells were further divided into B cell and T cell populations by gating for B220+ and CD3+ (middle panels). B cells (red) were B220+/CD3- and total T cells (black) were B220-/CD3+. CD3+ cells were subdivided (blue) into CD4+ (T helper) and CD8+ (T cytotoxic, right panels). Representative data from ventral group from blood sample from 6 week timepoint **(A)** and brain sample from 4 week timepoint **(B)**. **C, D.** Plasma cells were first identified via gating on CD45+ (left panels), then CD3- (middle panels), and then TACI+ and CD138+ double positivity (right panels, red). Representative data from ventral group from blood **(C)** and brain **(D)** samples from 4 week timepoint.

Supplementary Figure 6:

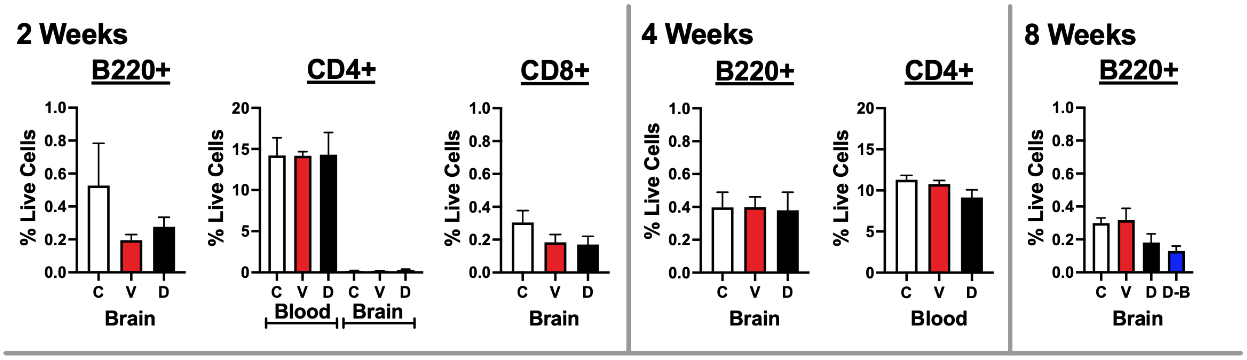

Supplementary Figure 6 legend: **Blood and brain lymphocytes.** Blood and half brain samples were collected at time of sacrifice at 2, 4, 6, and 8 weeks. At the 2 and 4-week timepoints, a single additional naïve age and sex-matched control mouse was added, providing two additional half-brains. At the 6-week timepoint, two additional naïve age and sex-matched control mice were added, providing four additional half-brains. A single spleen at each timepoint was also processed and stained for lymphocytes, as a positive staining control. Unstained cells were used

as a negative staining control. Cells were stained for CD4+ and CD8+ T cells (CD45+/CD3+/B220-), B220+ B cells (CD45+/CD3-/B220+), and CD138+/TACI+ plasma cells (CD45+/CD138+/TACI+). Flow cytometry results were quantified as a percentage of live cells. They are expressed as treatment group mean percentage. Error bars show standard errors of the means. At the 2 and 4-week timepoints, for blood, there were 3 samples from each immunized group and 4 samples from the control group and for brain, there were 3 half brains from each immunized group and 5 half brains from the control group. At the 8-week timepoint, there were 7 blood samples and half brains for the Control, Ventral (V) and Dorsal (D) unboosted mice and 6 blood samples and half brains for the Dorsal-boosted (D-B) mice, due to the death of a mouse in this group. The recording run malfunctioned for one of the 8-week control brain samples, which was excluded from the analyses. Dorsally-boosted mice were only represented at 6 and 8 weeks, as boosting occurred at 4 weeks. Results were tested for normality. For normally-distributed data, a 1-way ANOVA with Tukey's multiple comparison test was performed to determine differences between group means. For non-normally-distributed data, a Kruskal-Wallis 1-way ANOVA with Dunn's multiple comparison test was performed to determine differences between group means. No treatment group means were statistically different from the mean of the control group. Not all cell types were detected in all samples. For instance, plasma cells were not detected in the blood or brain samples at 2, 6, and 8 weeks and B220+ B cells were not detected in the brain samples at the 6-week timepoint.

Supplementary Table 2:

Cohort	Group	IFN-γ	IL-1β	IL-2	IL-4	IL-5	IL-6	IL-10	IL-12p70	KC/GRO	TNF-α
2-week	Control	41.9 ± 7.3	5.6± 0.9	17.7 ± 1.8	9.0 ± 1.0	78.8 ± 8.3	161.3 ± 13.9	X	X	91.1 ± 7.5	2.5 ± 0.1
	Ventral	36.2 ± 7.6	6.9 ± 0.5	15.9 ± 3.0	8.5 ± 0.6	70.5 ± 7.4	184.8 ± 16.3	X	X	*241.8 ± 34.3	*4.3 ± 0.4
	Dorsal	38.0 ± 6.3	6.7 ± 1.7	16.1 ± 1.0	9.4 ± 1.4	74.1 ± 13.4	219.1 ± 42.4	X	X	*234.7 ± 58.0	3.4 ± 0.3
4-week	Control	52.1 ± 6.6	6.0 ± 1.3	16.4 ± 1.6	8.5 ± 0.2	84.3 ± 12.4	180.6 ± 14.5	X	X	91.8 ± 5.4	2.1 ± 0.1
	Ventral	35.3 ± 5.4	6.0 ± 0.8	13.8 ± 2.2	9.0 ± 0.8	76.0 ± 10.0	213.1 ± 22.6	X	X	*151.2 ± 25.5	2.9 ± 0.4
	Dorsal	36.0 ± 6.7	6.5 ± 1.3	18.7 ± 2.2	8.8 ± 1.6	81.2 ± 15.9	153.3 ± 27.2	X	X	110.1 ± 5.0	2.2 ± 0.1
6-week	Control	40.1 ± 4.6	6.7 ± 0.9	15.0 ± 1.1	9.7 ± 0.7	73.4 ± 9.2	140.6 ± 10.9	X	X	82.1 ± 3.5	2.5 ± 0.1
	Ventral	54.1 ± 3.6	6.8 ± 0.7	16.7 ± 0.8	8.5 ± 0.9	69.0 ± 6.5	152.6 ± 26.1	X	X	107.3 ± 9.5	2.7 ± 0.0
	Dorsal	*11.0 ± 1.7	4.7 ± 0.8	11.5 ± 0.5	*5.4 ± 0.7	42.4 ± 5.3	131.8 ± 9.0	X	X	93.7 ± 8.6	2.5 ± 0.2
	Dorsal-boosted	*9.4 ± 4.0	7.2 ± 1.4	*11.0 ± 1.1	*5.8 ± 0.7	43.4 ± 5.5	160.6 ± 24.6	X	X	*247.5 ± 16.6	*3.6 ± 0.4
8-week	Control	5.9 ± 3.9	8.9 ± 0.7	4.3 ± 2.6	2.8 ± 1.2	29.2 ± 8.0	85.6 ± 13.9	X	X	132.3 ± 5.2	1.0 ± 0.4
	Ventral	0.5 ± 0.5	8.8 ± 0.7	3.9 ± 0.8	1.0 ± 0.4	26.1 ± 3.1	86.8 ± 9.0	X	X	178.1 ± 14.6	X
	Dorsal	1.9 ± 1.3	*12.2 ± 1.0	2.3 ± 0.6	2.7 ± 0.5	25.8 ± 2.8	103.7 ± 6.8	X	X	193.5 ± 18.1	X
	Dorsal-boosted	1.4 ± 0.9	*12.6 ± 1.4	2.1 ± 0.6	1.7 ± 0.3	31.3 ± 4.2	115.6 ± 14.4	X	X	*527.3 ± 82.9	1.3 ± 0.2
14-week	Control	17.9 ± 2.5	5.3 ± 0.5	6.2 ± 1.3	5.8 ± 0.6	61.3 ± 3.7	150.4 ± 9.9	X	X	67.4 ± 3.5	1.4 ± 0.1
	Ventral	25.2 ± 3.9	6.0 ± 0.4	6.1 ± 0.4	4.5 ± 0.4	51.2 ± 2.8	156.8 ± 10.0	X	X	106.6 ± 8.5	1.6 ± 0.2
	Dorsal	26.8 ± 4.3	7.0 ± 0.6	6.5 ± 0.5	6.0 ± 0.6	75.6 ± 9.3	*239.5 ± 22.1	X	X	*118.2 ± 10.4	*2.3 ± 0.2
	Dorsal-boosted	27.4 ± 4.7	7.7 ± 1.4	5.0 ± 0.3	*4.0 ± 0.5	55.2 ± 4.7	186.4 ± 20.1	X	X	*200.0 ± 18.5	2.6 ± 0.5

Supplementary Table 2 legend: **Brain cytokine levels.** A pro-inflammatory kit was used to evaluate cytokine levels for IFN-γ, IL-1β, IL-2, IL-4, IL-5, IL-6, IL-10, IL-12p70, KC/GRO (CXCL1), and TNF-α. Half brains were homogenized and spun down. Supernatants were tested for cytokines. Brains at 4 and 8-week timepoints were collected prior to boosting. Cytokine levels were quantified as pg per g whole brain tissue and

were averaged together per treatment group shown +/- standard errors of the means. There were 5 mice per group (Control, Ventral, Dorsal, and Dorsal-boosted (Dorsal-B)) at the 2, 4, and 6-week timepoints. Boosted mice only had samples from 6 weeks and beyond. There were 10 mice per group at the 8 and 14-week timepoints, with the exception of 9 dorsally-boosted mice at 8 weeks and 8 dorsally-induced mice at 14 weeks, due to mouse deaths in those groups. One ventrally-induced mouse in the 14-week group was excluded as an outlier (as confirmed by Prism software) due to values that were many magnitudes different from all other mice. Values which were below the reported threshold of detection for the assay are signified by an 'X'. Results were tested for normality. For normally-distributed data, a 1-way ANOVA with Tukey's multiple comparison test was performed to determine differences between group means. For non-normally-distributed data, a Kruskal-Wallis 1-way ANOVA with Dunn's multiple comparison test was performed to determine differences between group means. Treatment group means that were statistically different from the mean of the control group are signified by a '*'. *: $P < 0.05$.

Supplementary Figure 7:

A. 2-week timepoint

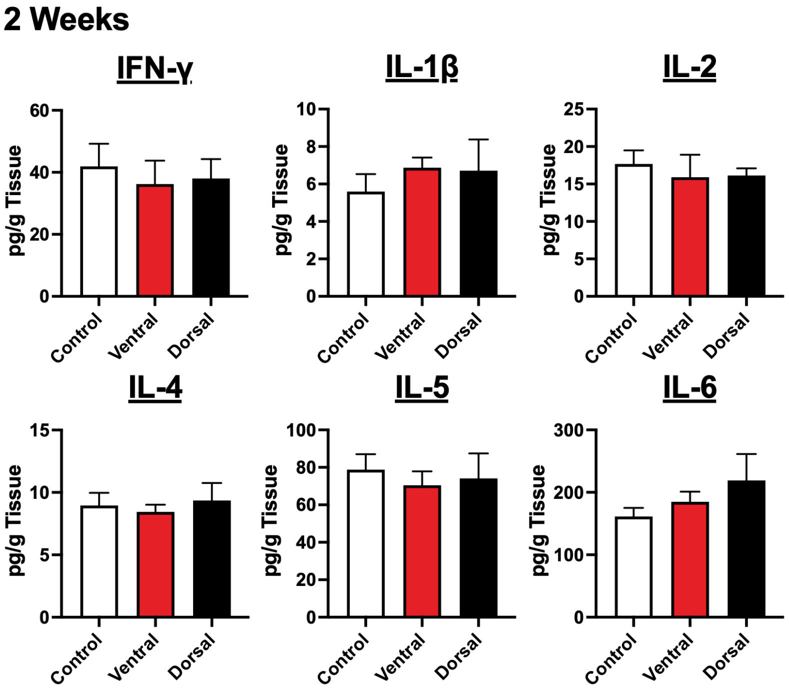

B. 4-week timepoint

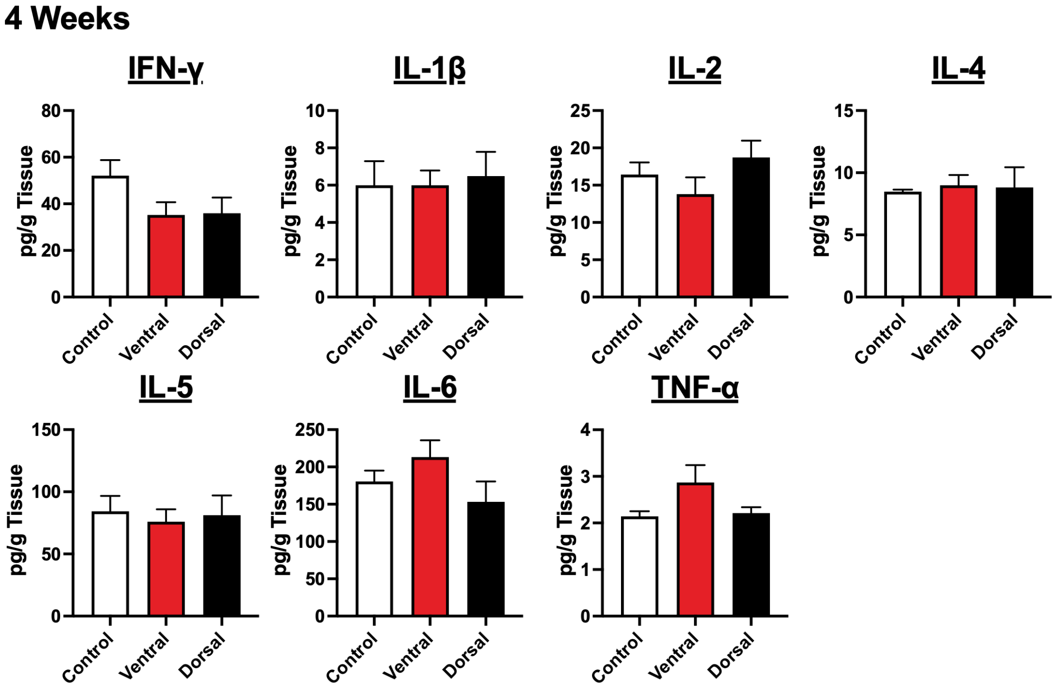

C. 6-week timepoint

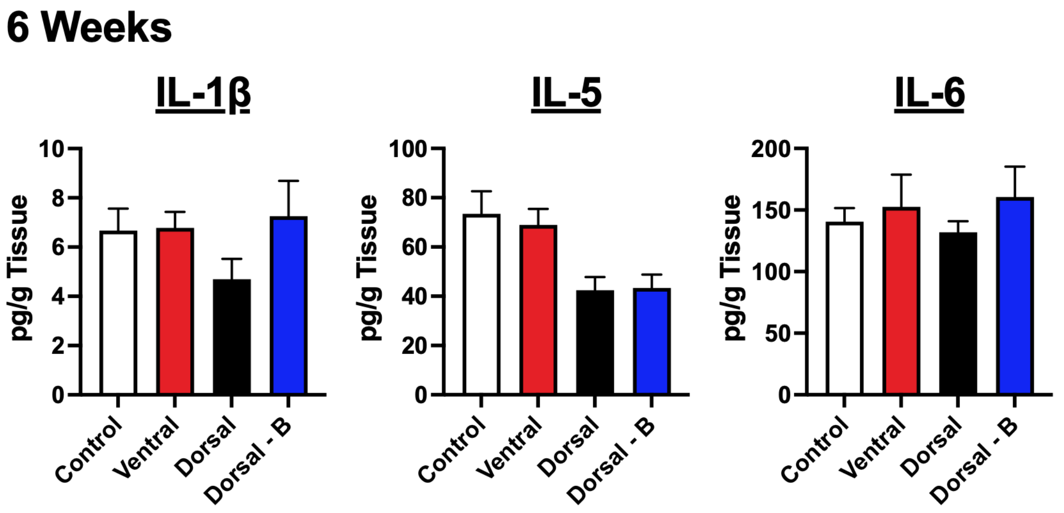

D. 8-week timepoint

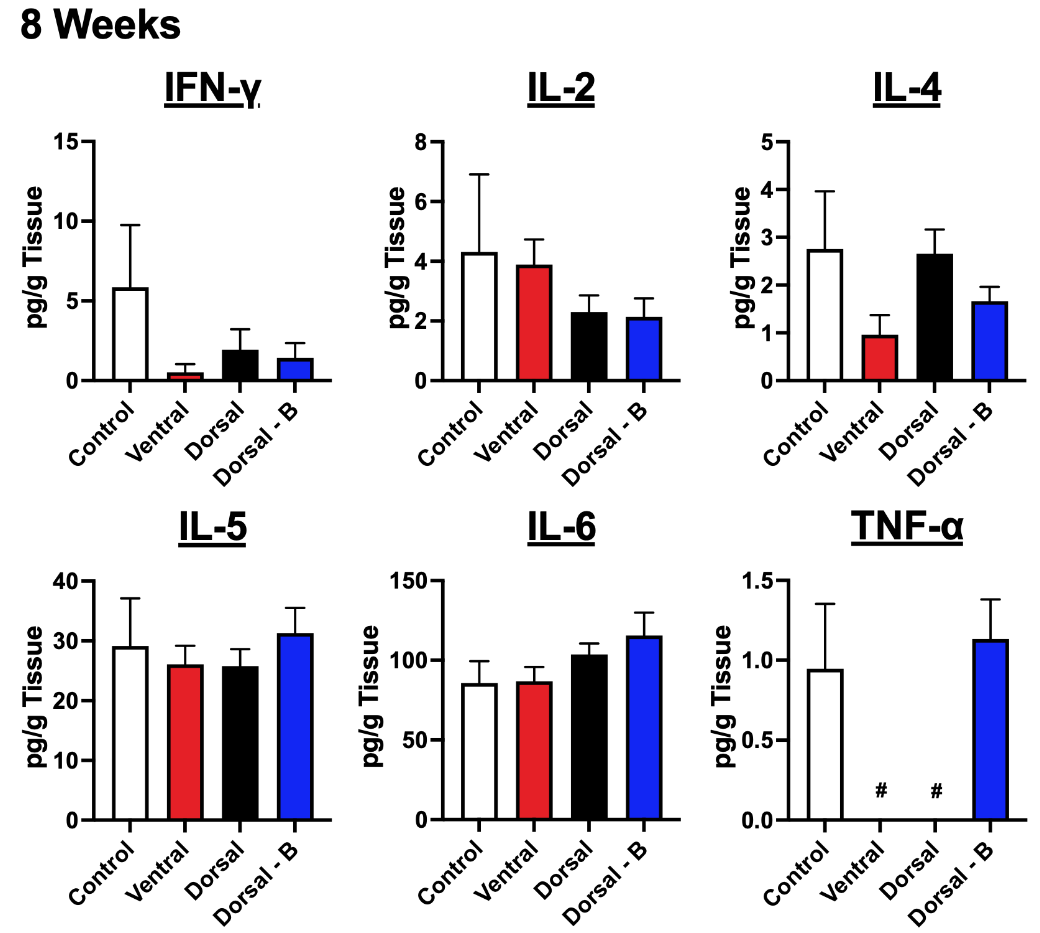

E. 14-week timepoint

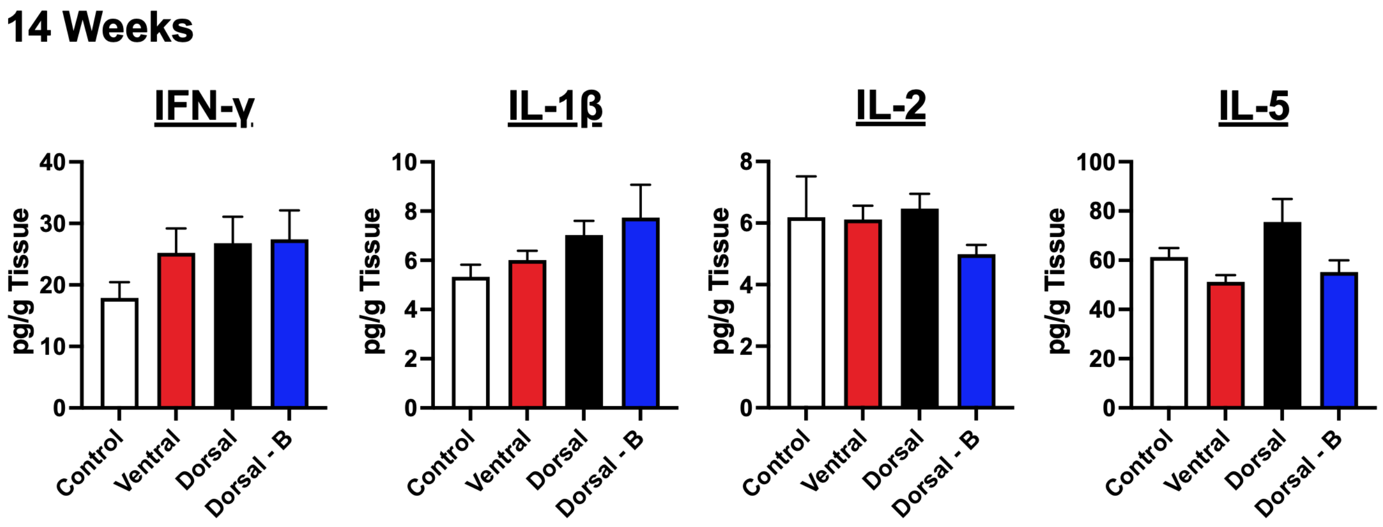

Supplementary Figure 7 legend: A pro-inflammatory kit was used to evaluate cytokine levels for IFN-γ, IL-1β, IL-2, IL-4, IL-5, IL-6, IL-10, IL-12p70, KC/GRO (CXCL1), and TNF-α. Half brains were homogenized and spun down. Supernatants were tested for cytokines. Brains at 4 and 8-week timepoints were collected prior to boosting. Cytokine levels were quantified as pg per g whole brain tissue. Error bars show standard errors of the means. Values which were below the reported threshold of detection for the assay (signified by 'X' in **Supplementary Table 2**, and '#' above) were excluded. There were 5 mice per group (Control, Ventral, Dorsal, and Dorsal-boosted (Dorsal-B)) at the 2, 4, and 6-week timepoints. Boosted mice only had samples from 6 weeks and beyond. There were 10 mice per group at the 8 and 14-week timepoints, with the exception of 9 dorsally-boosted mice at 8 weeks and 8 dorsally-induced mice at 14 weeks, due to mouse deaths in those groups. One ventrally-induced mouse in the 14-week group was excluded as an outlier (as confirmed by Prism software) due to values that were many magnitudes different from all other mice. Results were tested for normality. For normally-distributed data, a 1-way ANOVA with Tukey's multiple comparison test was performed to determine differences between group means. For non-normally-distributed data, a Kruskal-

Wallis 1-way ANOVA with Dunn's multiple comparison test was performed to determine differences between group means. No treatment group means were statistically different from the mean of the control.

Supplemental references:

1. Ding Y, Zhou Z, Chen J, et al. Anti-NMDAR encephalitis induced in mice by active immunization with a peptide from the amino-terminal domain of the GluN1 subunit. *J Neuroinflammation.* 2021;18(1):53.
2. Chen JW, Breckwoldt MO, Aikawa E, Chiang G, Weissleder R. Myeloperoxidase-targeted imaging of active inflammatory lesions in murine experimental autoimmune encephalomyelitis. *Brain.* 2008;131(Pt 4):1123-1133.
3. Planaguma J, Leypoldt F, Mannara F, et al. Human N-methyl D-aspartate receptor antibodies alter memory and behaviour in mice. *Brain.* 2015;138(Pt 1):94-109.
4. Barnes CA. Memory deficits associated with senescence: a neurophysiological and behavioral study in the rat. *J Comp Physiol Psychol.* 1979;93(1):74-104.
5. Attar A, Liu T, Chan WT, et al. A shortened Barnes maze protocol reveals memory deficits at 4-months of age in the triple-transgenic mouse model of Alzheimer's disease. *PLoS One.* 2013;8(11):e80355.
6. Olton DS. Mazes, maps, and memory. *Am Psychol.* 1979;34(7):583-596.
7. Labrousse VF, Costes L, Aubert A, et al. Impaired interleukin-1beta and c-Fos expression in the hippocampus is associated with a spatial memory deficit in P2X(7) receptor-deficient mice. *PLoS One.* 2009;4(6):e6006.
8. Wagnon I, Helie P, Bardou I, et al. Autoimmune encephalitis mediated by B-cell response against N-methyl-d-aspartate receptor. *Brain.* 2020;143(10):2957-2972.
9. Wang ME, Wann EG, Yuan RK, Ramos Alvarez MM, Stead SM, Muzzio IA. Long-term stabilization of place cell remapping produced by a fearful experience. *J Neurosci.* 2012;32(45):15802-15814.

10. Hall SC, Ballachey, E.L. A study of the rat's behavior in a field: a contribution to method in comparative psychology. *University of California Publications in Psychology.* 1932;6:1-12.
11. Cryan JF, Holmes A. The ascent of mouse: advances in modelling human depression and anxiety. *Nat Rev Drug Discov.* 2005;4(9):775-790.
12. Slattery DA, Cryan JF. Using the rat forced swim test to assess antidepressant-like activity in rodents. *Nat Protoc.* 2012;7(6):1009-1014.
13. Steru L, Chermat R, Thierry B, Simon P. The tail suspension test: a new method for screening antidepressants in mice. *Psychopharmacology (Berl).* 1985;85(3):367-370.
14. Can A, Dao DT, Terrillion CE, Piantadosi SC, Bhat S, Gould TD. The tail suspension test. *J Vis Exp.* 2012(59):e3769.
15. Deacon RM. Assessing nest building in mice. *Nat Protoc.* 2006;1(3):1117-1119.
